# Genetic Polymorphisms and Forensic Parameters of Thirteen X-Chromosome Markers in the Iraqi Kurdish Population

**DOI:** 10.1155/2024/9125094

**Published:** 2024-04-15

**Authors:** Ara K. Mohammad, Bahez Ismael, Khanzad Ahmed Ali, Balnd M. Albarzinji

**Affiliations:** Molecular Biology Department, Kurdistan Institution for Strategic Studies and Scientific Research (KISSR), Sulaymaniyah, Iraq

## Abstract

X-chromosome short tandem repeat (X-STR) tools are crucial in forensic genetics and human population fields. This study presents the development and validation of a multiplex STR system consisting of thirteen X-STR loci and amelogenin specific to the human X chromosome. The system was optimized and tested for species specificity, sensitivity, stability, and DNA mixture using 9947A female and 9948 male control genomic DNA. The amplified products of nine loci were sequenced to determine the correct amplicon length. Allele frequencies, forensic parameters, mean exclusion chance (MEC), linkage disequilibrium (LD), and allelic patterns were investigated using DNA samples from 225 (159 male, 66 female) unrelated Kurdish individuals who live in Sulaymaniyah province in the Kurdistan region of Iraq. The most informative locus in the Kurdish population was GATA172D05, while the least informative locus was DXS10164. The results demonstrated that the 13 X-STR system is highly polymorphic and sensitive for forensic DNA identification. Genetic distance-based clustering, metric multidimensional scaling (MDS), and correlation matrix were analyzed for 19 ethnic groups and populations. The phylogenetic tree showed that populations clustered according to their ethnogeographic relationships. The findings revealed genetic links between the Iraqi Kurds, Caucasians, Iraqi Arabs, United States (U.S.) ethnic groups, and Chinese populations.

## 1. Introduction

Short tandem repeat (STR) markers are specific regions on nuclear DNA that are highly polymorphic and used in forensic genetics to discriminate between DNA samples [1, 2]. In addition, many autosomal STR loci that have been recommended by the forensic community worldwide are ideal for paternity testing and human identifications [2, 3]. Nevertheless, using STRs from the sex chromosomes (X and Y) can also be valuable. The application of X-STRs plays an important role, particularly in complex cases where the analysis of autosomal loci is not informative [4, 5]. For example, the case of suspected half-sisters could be resolved by X-STR analysis as fathers will always pass on their single copy of the X chromosome to their daughters [6]. Moreover, because X chromosome STRs are polymorphic and easy to analyze, they are often used in population studies to evaluate the standard and rare alleles from a given population [6, 7]. In this work, a new reliable multiplex polymerase chain reaction (PCR) tool was developed and validated according to the Scientific Working Group on DNA Analysis Methods (SWGDAM) Validation Guidelines for DNA Analysis Methods [8]. This system can amplify 14 markers, including 13 STR loci on the human X chromosome (DXS9902, DXS10164, DXS7130, DXS7423, DXS8378, GATA172D05, DXS9898, DXS7424, GATA31E08, DXS6795, DXS981 (STRX1), DXS7132, and GATA144D04) and amelogenin for gender identification. This system was used to obtain genetic databases for population and forensic purposes and to further understand the genetic landscape of the Kurdish population in northern Iraq.

## 2. Materials and Methods

### 2.1. Primer Design

Primer pairs for 13 X-STR loci (DXS9902, DXS10164, DXS7130, DXS7423, DXS8378, GATA172D05, DXS9898, DXS7424, GATA31E08, DXS6795, DXS981 (STRX1), DXS7132, and GATA144D04) were designed using the publicly available software Primer3web version 4.1.0 (https://primer3.ut.ee/). A commonly used primer set for amelogenin, first published by Sullivan et al. [9], was used for gender identification. The primers were further checked for specificity using the National Center for Biotechnology Information (NCBI) (https://blast.ncbi.nlm.nih.gov/Blast.cgi). Unlabeled and labeled primer pairs were synthesized by the Microsynth AG company (Microsynth, Switzerland), and the forward primer of each labeled pair was fluorescently tagged at the 5′ end (FAM, ATTO532, ATTO550, or ATTO565) for the analysis by capillary electrophoresis. The primer details, fragment size, cytogenetic localization, and repeat motif are shown in Table 1.

Spectral calibration for Microsynth's dyes was performed using the Microsynth Matrix Standard (Mic-G5-Matrix-Std), which contains a mixture of five DNA fragments labeled with FAM, ATTO532, ATTO550, ATTO565, and Dyomics 630.

### 2.2. Sample Preparation

The work performed in this manuscript is part of a project approved by the Ethics Committee of the Kurdistan Institution for Strategic Studies and Scientific Research (KISSR). Buccal swab samples were collected with written informed consent from 225 healthy (66 female and 159 male) unrelated Kurds aged 18 and older from Sulaymaniyah province. Genomic DNA was extracted using the AddPrep Genomic DNA Extraction Kit (Add Bio, Korea) according to the manufacturer's protocol. Genomic DNA 9947A female (Qiagen, Germany) and 9948 male genomic DNA (MCLAB, USA) with known X-STR genotypes were purchased to be used as positive control. Purified DNA with numerical abnormalities was studied to evaluate the peak height ratio. The purity and concentration of DNA were determined using an Eppendorf Biophotometer Plus (Eppendorf, Germany).

### 2.3. PCR Condition and Sequencing

PCR conditions for the multiplex amplification were evaluated using gradient PCR with annealing temperatures of 55, 56, 57, 58, 59, and 60°C and the number of PCR cycles (26–30). All tests were based on 1 ng of 9948 control genomic DNA.

Multiplex PCR was carried out using Platinum® Multiplex PCR Master Mix (10 *μ*l) mixed with 1 *μ*l of DNA template, 6 *μ*l of the optimized primer mix (0.1 pmol), and 3 *μ*l of nuclease-free water to the volume of 20 *μ*l. Multiplex PCR amplification was performed using the following parameters: stage 1, activation of Platinum® Multiplex PCR Master Mix 95°C for 2 min; stage 2 (28 cycles), denaturation 95°C for 30 s, annealing 57°C for 60 s, and extension 72°C for 30 s; and stage 3, final extension 60°C for 30 min. The PCR amplification was performed using a Veriti® 96-Well Thermal Cycler (Applied Biosystems). The products were analyzed by capillary electrophoresis using the optimized analysis parameters for the ABI 3500 Prism® Genetic Analyzer. GeneScan 600 LIZ Size Standard v2.0 and Size-500 Plus were used as internal size standards for sizing DNA fragments.

PCR products were used for sequencing analysis. The samples were sequenced by Microsynth Seqlab GmbH (Microsynth, Germany) and Macrogen Inc. company (Macrogen, South Korea); the same PCR parameters described above were used with unlabeled primer pairs.

### 2.4. Species Specificity

Species specificity study was performed using DNA from nonhuman samples from common domestic animals (chicken, duck, pig, rabbit, and sheep) to evaluate the ability to detect genetic information from nonhuman biological samples. The extracted DNA amount of 2 ng was used with the 13 X-STR loci, and genomic male DNA 9948 (1 ng) was amplified as a positive control.

### 2.5. Sensitivity and Stability

A sensitivity study was conducted using serial dilutions of 9948 control genomic DNA to evaluate the minimum amount of DNA required to obtain reliable results. Amplification of control DNA was carried out in triplicate with the following quantities: 2.5, 1.25, 0.6, 0.3, 0.1, and 0.05 ng in a final volume of 20 *μ*l using the optimal PCR parameters.

The stability study was conducted by adding different concentrations of three common types of PCR inhibitors: ethanol, isopropanol, and ethylenediaminetetraacetic acid (EDTA). The control DNA template was 1 ng, and the inhibitor concentrations were as follows: 9.6, 4.8, 2.4, 1.2, and 0.6% ethanol; 9.9, 4.9, 2.4, 1.2, and 0.6% isopropanol; and 5, 2.5, 1.25, 0.625, 0.31, and 0.15 mM EDTA in a final PCR volume of 20 *μ*l.

### 2.6. Mixture Study

Female 9947A and male 9948 DNA samples were prepared in triplicates at 1 : 1, 2 : 1, and 1 : 2 ratios to evaluate the performance of the in-house tool for DNA mixture detection. The total amount of DNA was 0.5 ng in a final volume of 20 *μ*l of the PCR.

Extracted DNA from female and male samples were quantified using Eppendorf Biophotometer Plus. Female-male mixtures at different ratios (1 : 1, 3 : 1, 5 : 1, 8 : 1, 1 : 3, 1 : 5, and 1 : 8) were prepared and amplified in triplicates using 1 ng in the final volume of 20 *μ*l of PCR.

### 2.7. Data Analysis

The allele frequencies of the 13 X-STR loci for the female and male data were calculated using StatsX v2.0 [10]. Allelic patterns were calculated using genetic analysis in Excel (GenAIEx 6.5) [11]. The linkage disequilibrium (LD) and forensic statistical parameters, including gene diversity (GD), polymorphism information content (PIC), power of discrimination (PD), and match probability (PM), were calculated using the online tool STR Analysis for Forensics (STRAF 2.1.5) [12]. The combined PD, male and female, and mean exclusion chance (MEC_Kruger_, MEC_Kishida_, MEC_Desmarais_, and MEC_Desmarais duo_) were calculated according to Hauston [13] using the StatsX v2.0. The sequence data were viewed and analyzed using Chromas (version 2.6.6) DNA sequence analysis software. The phylogenetic tree was constructed from allele frequency using POPTREE2 software [14] and visualized by an Interactive Tree Of Life (iTOL) v5 [15]. The tree was performed based on data from seven loci of 19 populations using neighbor-joining phylogeny and fixation index (*F*_ST_ (uncorrected)) distance. The metric multidimensional scaling plot (MDS) was generated using *F*_ST_ values by Kamakura's Analytic Tools for Excel [16]. The correlation matrix was used to investigate genetic comparisons between the populations based on *F*_ST_ values using the R statistical software version 4.3.2 and RStudio [17, 18].

## 3. Results

### 3.1. Multiplex Design

Thirteen X-STR and amelogenin were selected based on the following criteria: (1) their locations to cover the entire X chromosome (Figure S1), four loci are on the short arm of the X chromosome (DXS8378, DXS9902, DXS6795, and GATA144D04), two loci are on the centromere (DXS10164 and DXS7132), and seven loci are on the long arm (DXS981, DXS9898, DXS7424, GATA172D05, DXS7130, GATA31E08, and DXS7423); (2) the loci that are with high polymorphisms were selected; (3) suitable for designing multiplex primers with amplicon sizes below 340 bp; and (4) spaces between the markers on the same dye channel.

PCR conditions, gradient PCR (55–60°C), and number of cycles (26–30) were used to evaluate the reaction conditions (Figure S2). The 13 X-STR loci and amelogenin were successfully optimized and amplified in a single PCR. The amplification of the in-house X-STR loci was set up using the annealing temperature of 57°C and 28 cycles. Allele designations (bins and panels) were created by comparing the female 9947A and the male 9948 genomic control DNA (Table S1). Electropherograms of the DNA profiles are shown in Figures S3 and S4. The PCR products of 9 loci (DXS6795, DXS7130, DXS7424, GATA172D05, GATA31E08, DXS10164, DXS9898, DXS981, and DXS9902) were sequenced to confirm the exact length of the amplicons using different male DNA templates (Figure S5).

### 3.2. Sensitivity and Stability

Different concentrations of DNA (9948 male) ranging from 2.5 to 0.05 ng were amplified to determine the minimum amount of DNA sample from which a complete profile can be generated (Figure S6). The optimal amount of DNA required to obtain a reliable profile was 1 ng using 28 PCR cycles. However, satisfactory results were obtained using a DNA amount of 0.125 ng without increasing the PCR cycles (Figure S7). Allele dropouts of DXS7130, DXS8378, DXS9898, and DXS981 were observed at 0.05 ng of DNA.

The stability test was performed using different concentrations of three inhibitory substances (ethanol, isopropanol, and EDTA). The results revealed that complete DNA profiles were obtained from 1 ng of 9948 DNA samples until up to 1.25% ethanol and isopropanol and 0.625 mM EDTA in the final 20 *μ*l PCR volume. Allele dropouts were observed at higher concentrations of 2.4% ethanol and isopropanol and 1.25 mM EDTA. The entire amplification failure was obtained when the concentrations increased to ethanol (4.8%), isopropanol (4.9%), and EDTA (2.5 mM) in the final reaction volume (Figure S8).

### 3.3. Species Specificity

The species specificity test was performed using DNA extracted from five different animals: chicken, duck, pig, rabbit, and sheep. The results showed no specific peaks at all loci were observed (Figure S9). This result demonstrated that the 13 X-STR tool is suitable for human identity testing.

### 3.4. Mixture Study

Mixtures arise when two or more DNA sources contribute to a single sample. Therefore, female-male DNA mixtures at different ratios, 9947A female and 9948 male (1 : 1, 2 : 1, and 1 : 2), extracted DNA female-male (1 : 1, 3 : 1, 5 : 1, 8 : 1, 1 : 3, 1 : 5, and 1 : 8) were studied (Figure S11). The results revealed no allele dropout at 2 : 1 and 1 : 2 of control DNA mixtures. In addition, DNA mixtures of the purified samples were identified even at 1 : 8 and 8 : 1 ratios; however, the height of the peaks was proportional to the amount of DNA. Increasing the PCR cycles and the amount of DNA might improve the sensitivity of detecting the mixture samples. The results suggested that the 13 X-STR tool was suitable for detecting DNA mixture samples with two individuals.

### 3.5. Peak Height Ratio

DNA samples with numerical abnormalities on the X chromosome (X0, XXY, and XXX) were analyzed; chromosomal anomalies were confirmed in these samples using commercial kits. Turner syndrome, also called 45, X0, is when females with this disorder have 45 chromosomes instead of 46; they lack one X chromosome. Klinefelter syndrome, also known as 47, XXY, is when males with this disorder have one extra copy of the X chromosome. Triple X syndrome, also called trisomy X syndrome, is when females in this condition inherit an extra X chromosome. The peak height ratio was calculated by dividing the peak height of a lower relative fluorescence unit (RFU) value by the peak height of a higher RFU value. The results, shown in Figure S10, revealed imbalanced peak heights with an average ratio of less than 70% at eight loci and one triallelic locus (DXS7130) in the triple X sample. Seven loci were biallelic and imbalanced X, Y amelogenin with a peak height ratio of less than 60% (52.6%) in the XXY sample, and monosomy in the X0 sample was obtained. The results demonstrated that the 13 X-STR loci could distinguish between monosomic and trisomic states of the X chromosome, indicating that this method is reliable for diagnosing sex aneuploidies.

### 3.6. Allele Frequency and Forensic Parameter

**Figure 1:**
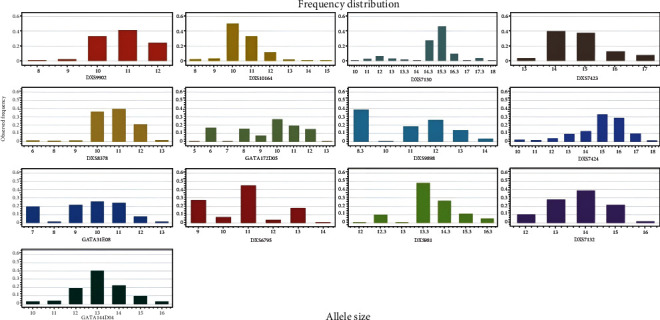
Plots showing the distribution of allele frequencies of the 13 X-STR loci using pool data of the Kurds from Sulaymaniyah City, Iraq. The horizontal axis (*X*-axis) represents the allele size, and the vertical axis (*Y*-axis) represents the observed frequency.

Based on the 13 X-STR loci, the X chromosome data were analyzed in the Kurdish population samples, males and females, from Sulaymaniyah province in northern Iraq. The results can be found in Supplementary Tables S2 and S3. Allelic frequencies for male, female, and pool samples were calculated, and the results are shown in Table S4. The distribution plots of the pool allele frequencies are presented in Figure 1.

Forensic statistical parameters were calculated using the STRAF online tool. The results showed that the highest PIC and GD were observed at the GATA172D05 locus in the female (PIC = 0.7891, GD = 0.8218) and male (PIC = 0.7878, GD = 0.8185) samples. The lowest PIC and GD were found at the DXS10164 locus in the female (PIC = 0.5314, GD = 0.5859) and male (PIC = 0.6811, GD = 0.6516) samples, as shown in Supplementary Tables S5 and S6.

**Figure 2:**
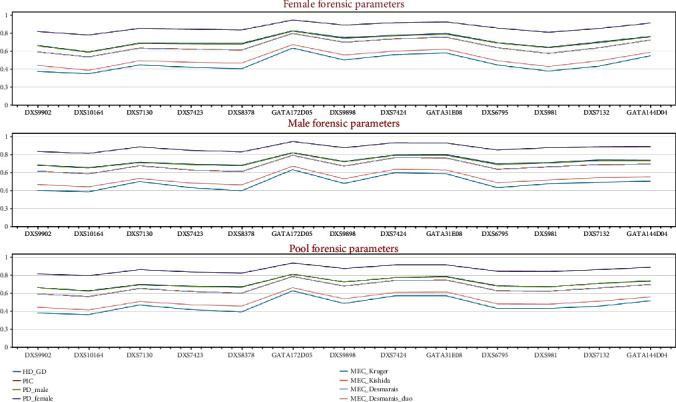
Graph of forensic parameters and mean exclusion chance distribution of the 13 X-STR loci using female, male, and pool data.

The combined power of discrimination for the Kurdish male and female was calculated using the 13 X-STR loci, and the results were 0.999999933 and 0.99999999999828, respectively. The combined MEC_Kruger_, MEC_Kishida_, MEC_Desmarais_, and MEC_Desmarais duo_ were 0.999815, 0.999999523, 0.999999524, and 0.9999492, respectively (Table S7). The GATA172D05 locus had the most significant overall MEC value, while the lowest MEC value was at the DXS10164 locus (Figure 2). These results indicated that the 13 X-STR loci can be used to establish a DNA database for a particular population.

A total number of 93 alleles was observed for the 13 X-STR loci. The number of different alleles at each locus varied, ranging from 12 for the DXS7130 locus to 5 for the DXS9902, DXS7132, and DXS7423 loci (Table S7). In addition, the GATA172D05 locus had the highest number of effective alleles (Ne) (female Ne = 5.425, male Ne = 5.360). In contrast, the locus DXS10164 had the lowest Ne for the female (Ne = 2.389) and male (Ne = 2.837) samples, as shown in Table S8.

### 3.7. Linkage Disequilibrium

The exact pairwise test of LD for all pairs of loci was tested by the STRAF 2.1.5 online tool using female and male data separately. In this study, 78 pairwise comparisons were performed. The results are shown in Supplementary Tables S9 and S10. After applying Bonferroni's correction (*p* < 0.0003, male) and (*p* < 0.0007, female), a significant association was found in one pair of loci, which was between the DXS7130 and DXS981 loci (*p* = 0.0001) in the female data. There was no statistically significant LD in the male data.

### 3.8. Population Study

**Figure 3:**
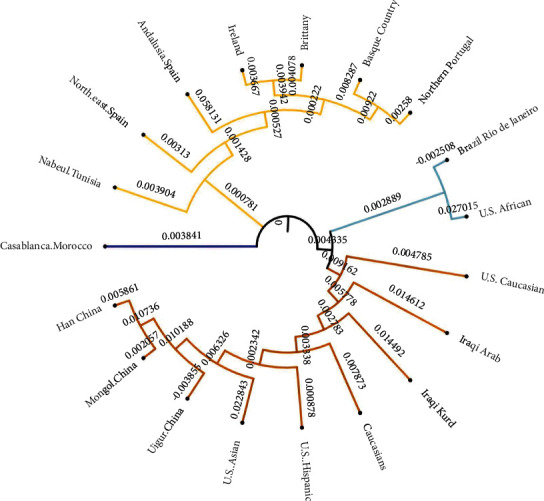
Phylogenetic tree generated using POPTREE2 tool based on *F*_ST_ values of 7 X-STR loci in the Iraqi Kurds and 18 other ethnic groups and populations.

In the present study, allele frequency data from seven loci were used to compare genetic variations among the Iraqi Kurd and 18 other ethnic groups and populations, including Iraqi Arab, U.S. African, U.S. Caucasian, U.S. Hispanic, U.S. Asian, Han China, Uigur China, Mongol China, Casablanca Morocco, northeast Spain, Brittany, Ireland, northern Portugal, Andalusia Spain, Basque Country, Caucasians, Nabeul Tunisia, and Brazil Rio de Janeiro [19–28]. Four subpopulations were obtained, as shown in Figure 3. The inferred subpopulations were as follows: cluster 1: Iraqi Kurds, Caucasians, Iraqi Arabs, U.S. Caucasians, U.S. Hispanics, U.S. Asians, and Chinese (Uigur, Mongol, and Han); cluster 2: northern Portugal, Basque Country, Brittany, Ireland, Andalusia Spain, northeast Spain, and Nabeul Tunisia; cluster 3: Brazil Rio de Janeiro and U.S. African; and cluster 4: Casablanca Morocco.

**Figure 4:**
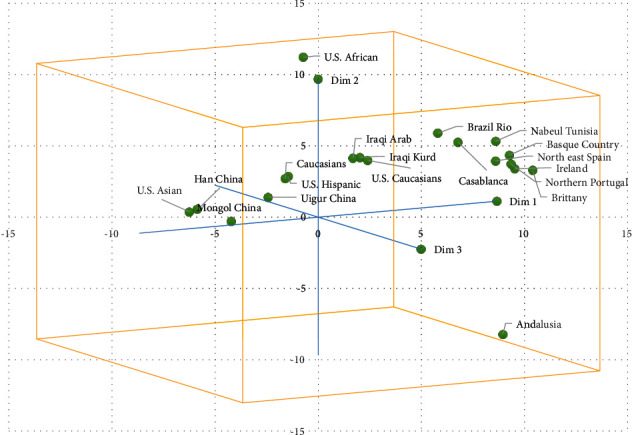
A metric multidimensional scaling analysis based on the genetic distance values (*F*_ST_) of the Iraqi Kurdish groups and 18 other populations.

The metric multidimensional scaling (MDS) was generated to obtain the relationships among samples from the Kurdish ethnic group and 18 other populations. The MDS results showed the level of similarity between the populations (Figure 4). The upper right quadrant had two groups: the first group included Iraqi Arabs, Iraqi Kurds, and U.S. Caucasians; the second group was Brazil Rio de Janeiro, Nabuel Tunisia, Basque country, northeast Spain, Ireland, northern Portugal, and Brittany. The upper left quadrant had six populations, including Caucasians, U.S. Hispanics, Uigur China, Mongol China, Han China, and U.S. Asian, while the U.S. African and Andalusia were separated from all ethnic groups outside the clusters.

**Figure 5:**
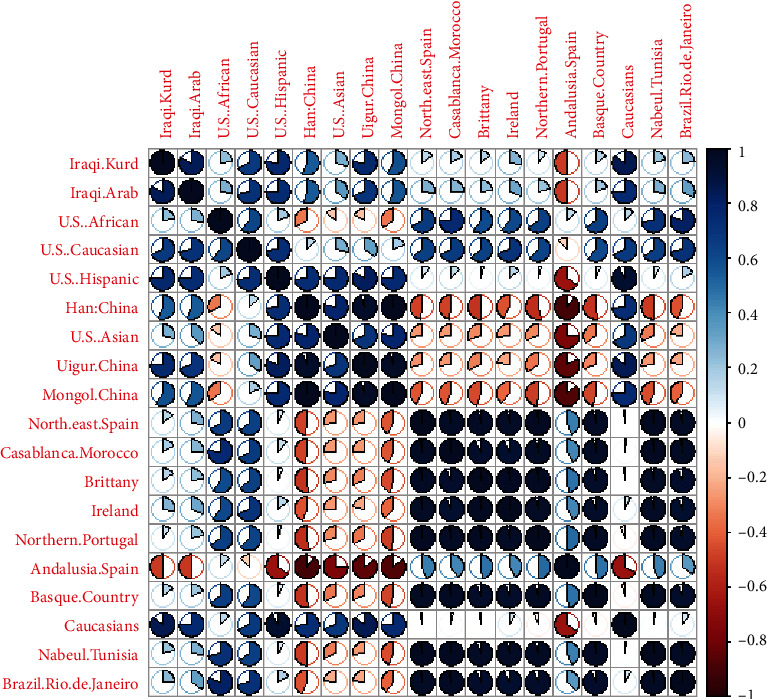
Pearson's correlation coefficient matrix measuring the genetic distances among 19 ethnic groups using the corrplot package in R statistical software.

The genetic correlation matrix was constructed to depict the association between genetic differentiations in these ethnic groups and populations (Figure 5). The results revealed a strong relationship between Iraqi Kurds, Caucasians, Iraqi Arabs, U.S. Hispanics, and Uigur China. In contrast, the negative relationship was with northern Portugal, Brittany, and northeast Spain.

The evolutionary relationship studies revealed that the Iraqi Kurds are more genetically related to the Iraqi Arabs, Caucasians, U.S. Caucasians, U.S. Hispanics, and Chinese populations.

## 4. Discussion

This study investigated allele frequencies and forensic parameters of the Kurdish population in Sulaymaniyah province using noncommercial kit markers. Despite the limited number of loci in this panel, informative results were obtained by analyzing 13 X-STRs and amelogenin, which have been added to the Kurdish genetic data. These findings can be utilized in forensic DNA and population genetic studies.

ChrX-STR.org2.0 (https://www.chrx-str.org/xdb/index.jsf) is a website that provides databases of chromosome X-STRs. However, some markers have limited genetic data for a few populations, such as DXS7424, DXS6795, DXS10164, DXS7130, and GATA144D04. In addition, the limited availability of the X-chromosome STR database resulted in a reduced number of genetic loci used to construct the phylogenetic tree.

Previous research on the Kurdish X-STRs used the Investigator Argus X-12 QS kit, which consists of twelve loci organized into four linkage groups [29]. In contrast, our in-house tool includes 13 loci, with ten of them not present in the Investigator Argus X-12 QS kit. Previous studies have developed in-house X-STRs in Iraq [19, 30]. However, they have selected different sets of X-chromosome loci compared to our work. In addition, DNA samples from Iraqi Arab males in Baghdad City were only examined. Other studies have reported developing and validating new X-STR assays utilizing different combinations of the X-STR loci to obtain genetic information from particular populations and to investigate the forensically relevant parameters [31–35]. The present study found that the highest and lowest GD and PIC in the Kurdish population were at the GATA172D05 and DXS10164 loci, respectively. The same locus (GATA172D05) was found to be the most informative in the Murcia population in Spain [36]. The least informative locus (DXS10164) was also determined in the Chinese Uygur population [37].

Using X-chromosome STR loci, commercial and noncommercial kits may serve as an efficient complementary tool to autosomal STR, Y-STR, and mitochondrial DNA markers in forensic investigations; this is particularly applicable in paternity cases where the available information is uncertain.

## 5. Conclusions

In this study, a specific PCR system was designed for the human X chromosome. Hierarchical tree and population comparisons revealed clustering based on ethnogeographic relationships. The findings demonstrated that this X-chromosome system is reliable and effective in analyzing numerical X-chromosome abnormalities and establishing genetic databases. However, increasing the loci will lead to more accurate population and forensic genetic studies. Furthermore, incorporating loci with more alleles into the in-house X-STR will enhance the results and develop a robust tool suitable for identification in complex forensic cases.

## Figures and Tables

**Table 1:** Table showing locus name and characteristics of the 13 X-STR loci and amelogenin included in the PCR system.

Locus	Cytogenetic localization	Repeat motif	Allele range	Primer sequence (5′-3′)	Dye	Fragment size (bp)	TM (°C)
AMEL	X: p 22.1-22.3 Y: p 11.2	—	X, Y	F-CCCTGGGCTCTGTAAAGAATA R-ATCAGAGCTTAAACTGGGAAGCTG	FAM	106-112	59 60.9
DXS9902	p 22.2	ATCT	7-13	F-GTACTGTTCTTGTAACTTTTCTGTCG R-CAG GAG TAT GGG ATCACCAGTGA	FAM	118-148	63.1 66.3
DXS10164	Centromere	ATTCT	7-15	F-GGCAGTATTAATCAAAACAGAATGG R-TGAACGGAATGTACTTTTCCAA	FAM	180-224	55.7 56.6
DXS7130	q 24	TATC	10-18	F-TCCTTCAATTGCCCCATCGATAG R-GTGCCCAATGAATGCCTCCATA	FAM	265-302	62 62.3
DXS7423	q 28	TCCA	12-18	F-GCCACTGGGGACATGTGAATGG R-CACCCAGATTTCCTCCCCA	FAM	302-330	66.9 64.7
DXS8378	p 22.31	CTAT	6-14	R-GCGACAAGAGCGAAACTCCA F-GCTCCTGGCAGGTCACTATC	ATTO532	80-118	62.9 62.3
GATA172D05	q 23	TAGA	4-13	F-TACTGTTTAACCAGTACAAAGTT R-TCTATAGATATAGGTATTGATATAGC	ATTO532	124-164	55.6 51.1
DXS9898	q 21.31	TATC	8-15	F-CGAGCACACCTACAAAAGCT R-TCGATTAGGTTCAGTTCCCA	ATTO532	183-219	59 58.5
DXS7424	q 22.1	TAA	8-19	F-GAGTCCAGGAATTCAAGGCCT R-TACAGCTAAGAAGAATCCCGCA	ATTO550	100-138	62.7 59.7
GATA31E08	q 27.1	AGAT	7-13	F-TGTTTATCATACTAGATATAGATA R-AGATACATGTATGTATGCTCACT	ATTO550	150-182	53.4 57.6
DXS6795	p 22.11	AAT	9-17	F-CTGGTCCAACTGATGCACAC R-GAAATGCATCCATCCCCTAA	ATTO550	214-249	61.2 57.4
DXS981 (STRX1)	q 13.1	TATC	12-17	F-CTTCTCCAGCACCCAAGGAAGTCA R-CTCCTTGTGGCCTTCCTTAAATGG	ATTO550	307-334	66.8 63.5
DXS7132	Centromere	TCTA	11-17	F-ATAAATCCCCTCTCATCTATCTGAC R-ACTCCTGGTGCCAAACTCTA	ATTO565	128-158	57.7 60.7
GATA144D04	p 11.23	CTAT	9-17	F-AAGATCTGCCAAGCCAGAAG R-CTTGCAGTGAGGGACAGAGA	ATTO565	225-262	59.6 62.1

## Data Availability

Supplementary data associated with this project can also be found in the supplementary materials.

## Supplementary Materials

Supplementary Materials The following are available online: Figure S1: name and location of the in-house 13 X-STR loci and amelogenin on the X chromosome. Figure S2: evaluation of the PCR conditions for the 13 X-STR multiplex system using (A) gradient PCR with annealing temperatures of 55, 56, 57, 58, 59, and 60°C and (B) number of PCR cycles (26, 27, 28, 29, and 30). All tests were based on 1 ng of 9948 control genomic DNA. Figure S3: electropherogram of the female control DNA 9947A using the 13 X-STR loci. Figure S4: electropherogram of the male genomic control DNA 9948 using the 13 X-STR loci. Figure S5: DNA sequences of 9 amplicons (DXS6795, DXS7130, DXS7424, GATA172D05, GATA31E08, DXS10164, DXS9898, DXS981, and DXS9902) using PCR products of male DNA samples. Figure S6: electropherograms show amplification of the control DNA (9948 male) with the following quantities: 2.5, 1.25, 0.6, 0.3, 0.1, and 0.05 ng to test the sensitivity of the 13 X-STR tool. Figure S7: electropherogram shows the complete profile of the control DNA 9948 male using a DNA amount of 1.25 ng. Figure S8: electropherograms show amplification of the 9948 male control DNA template (1 ng) with inhibitory substances at different concentrations: (A) EDTA (5, 2.5, 1.25, 0.625, 0.31, and 0.15 mM), (B) ethanol (9.6, 4.8, 2.4, 1.2, and 0.6%), and (C) isopropanol (9.9, 4.9, 2.4, 1.2, and 0.6%) in a final volume of 20 *μ*l of PCR reaction. Figure S9: electropherograms show amplification of nonhuman DNA samples from common domestic animals (chicken, duck, pig, rabbit, and sheep) to evaluate the species specificity study. Figure S10: electropherograms show amplification of DNA samples with numerical abnormalities on the X chromosome: (A) Turner syndrome (45, X0), (B) Klinefelter syndrome (47, XXY), and (C) triple X syndrome (trisomy X). Figure S11: electropherograms show amplification of female-male DNA mixtures at different ratios: (A) 9947A female and 9948 male control DNA (1 : 1, 2 : 1, and 1 : 2) and (B) extracted DNA female-male mixtures (1 : 1, 3 : 1, 5 : 1, 8 : 1, 1 : 3, 1 : 5, and 1 : 8). Supplementary Table S1: table shows genetic DNA profiles and the alleles found at each locus of control DNA 9947A female and 9948 male using the in-house 13 X-STR markers. Supplementary Table S2: genetic profiles of the 13 X-STR loci were obtained from 159 unrelated Kurd males. Supplementary Table S3: genetic profiles of the 13 X-STR loci were obtained from 66 unrelated Kurd females. Supplementary Table S4: allele frequencies of the 13 X-STR loci using female (F), male (M), and pool (P) data. Supplementary Table S5: forensic parameters, gene diversity (GD), polymorphism information content (PIC), match probability (PM), power of discrimination (PD), observed heterozygosity (Hobs), and power of exclusion (PE) were calculated for the in-house 13 X-STR loci in the Kurdish female using the STRAF online tool. Supplementary Table S6: forensic parameters, gene diversity (GD), polymorphism information content (PIC), match probability (PM), and power of discrimination (PD) were calculated for the in-house 13 X-STR loci in the Kurdish male using the STRAF online tool. Supplementary Table S7: combined power of discrimination, mean of exclusion (MEC), and combined MEC of the 13 X-STR loci using the StatsX v2.0. Supplementary Table S8: number of alleles, number of effective alleles, information index, diversity, and unbiased diversity were determined by locus for the male and female Kurds using GenAIEx 6.5. Supplementary Table S9: pairwise *p* values of linkage disequilibrium test for all pairs of loci using 66 female samples from the Kurdish population. Supplementary Table S10: pairwise *p* values of linkage disequilibrium test for all pairs of loci using 159 male samples from the Kurdish population.
