# Supplementary material for: Genetic Polymorphisms and Forensic Parameters of Thirteen X-Chromosome Markers in the Iraqi Kurdish Population
Source: J Nucleic Acids. 2024 Apr 15;2024:9125094. doi: 10.1155/2024/9125094 (PMC11074882; doi:10.1155/2024/9125094)
Supplement: Supplementary Materials — The following are available online: Figure S1: name and location of the in-house 13 X-STR loci and amelogenin on the X chromosome. Figure S2: evaluation of the PCR conditions for the 13 X-STR multiplex system using (A) gradient PCR with annealing temperatures of 55, 56, 57, 58, 59, and 60°C and (B) number of PCR cycles (26, 27, 28, 29, and 30). All tests were based on 1 ng of 9948 control genomic DNA. Figure S3: electropherogram of the female control DNA 9947A using the 13 X-STR loci. Figure S4: electropherogram of the male genomic control DNA 9948 using the 13 X-STR loci. Figure S5: DNA sequences of 9 amplicons (DXS6795, DXS7130, DXS7424, GATA172D05, GATA31E08, DXS10164, DXS9898, DXS981, and DXS9902) using PCR products of male DNA samples. Figure S6: electropherograms show amplification of the control DNA (9948 male) with the following quantities: 2.5, 1.25, 0.6, 0.3, 0.1, and 0.05 ng to test the sensitivity of the 13 X-STR tool. Figure S7: electropherogram shows the complete profile of the control DNA 9948 male using a DNA amount of 1.25 ng. Figure S8: electropherograms show amplification of the 9948 male control DNA template (1 ng) with inhibitory substances at different concentrations: (A) EDTA (5, 2.5, 1.25, 0.625, 0.31, and 0.15 mM), (B) ethanol (9.6, 4.8, 2.4, 1.2, and 0.6%), and (C) isopropanol (9.9, 4.9, 2.4, 1.2, and 0.6%) in a final volume of 20 μl of PCR reaction. Figure S9: electropherograms show amplification of nonhuman DNA samples from common domestic animals (chicken, duck, pig, rabbit, and sheep) to evaluate the species specificity study. Figure S10: electropherograms show amplification of DNA samples with numerical abnormalities on the X chromosome: (A) Turner syndrome (45, X0), (B) Klinefelter syndrome (47, XXY), and (C) triple X syndrome (trisomy X). Figure S11: electropherograms show amplification of female-male DNA mixtures at different ratios: (A) 9947A female and 9948 male control DNA (1 : 1, 2 : 1, and 1 : 2) and (B) extracted DNA female-ma [file 9125094.f1.zip › Supplementary Figures (1).docx]

**Supplementary Figures**

**Figure S1:** Name and location of the in-house 13 X-STR loci and amelogenin on the X-chromosome.

**Figure S2:** Evaluation of the PCR conditions for the 13 X-STR multiplex system using (A) gradient PCR with annealing temperatures of (55, 56, 57, 58, 59, and 60 ° C) and (B) number of PCR cycles (26, 27,28,29, and 30). All tests were based on 1ng of 9948 control genomic DNA.

**Figure S3:** Electropherogram shows the alleles (al) and size (sz) of the 13 X-STR loci and amelogenin using female control DNA 9947A.

**Figure S4:** Electropherogram shows the alleles (al) and size (sz) of the 13 X-STR loci and amelogenin using control DNA 9948.

**Figure S5:** DNA sequences of 9 amplicons (DXS6795, DXS7130, DXS7424, GATA172D05, GATA31E08, DXS10164, DXS9898, DXS981, DXS9902) using PCR products of male DNA samples.

**Figure S6:** Electropherograms show amplification of the control DNA (9948 male) with the following quantities: 2.5, 1.25, 0.6, 0.3, 0.1, and 0.05 ng to test the sensitivity of the 13 X-STR tool.

**Figure S7:** Electropherogram shows the complete profile of the control DNA 9948 male using DNA amount of 1.25 ng.

**Figure S8:** Electropherograms show amplification of the 9948 male control DNA template (1 ng) with inhibitory substances at different concentrations: (A) EDTA (5, 2.5, 1.25, 0.625, 0.31, 0.15 mM), (B) Ethanol (9.6, 4.8, 2.4, 1.2, and 0.6 %), and (C) Isopropanol (9.9, 4.9, 2.4, 1.2, 0.6 %) in a final volume of 20 µl of PCR reaction.

**Figure S9:** Electropherograms show amplification of non-human DNA samples from common domestic animals (chicken, duck, pig, rabbit, and sheep) to evaluate the species specificity study.

**Figure S10:** Electropherograms show amplification of DNA samples with numerical abnormalities on the X chromosome (A) Turner syndrome (45, X0), (B) Klinefelter syndrome (47, XXY), and (C) Triple X syndrome (Trisomy X).

**Figure S11:** Electropherograms show amplification of female-male DNA mixtures at different ratios, (A) 9947A female and 9948 male control DNA (1:1, 2:1, and 1:2), and (B) extracted DNA female-male mixtures (1:1, 3:1, 5:1, 8:1, 1:3, 1:5, and 1:8).


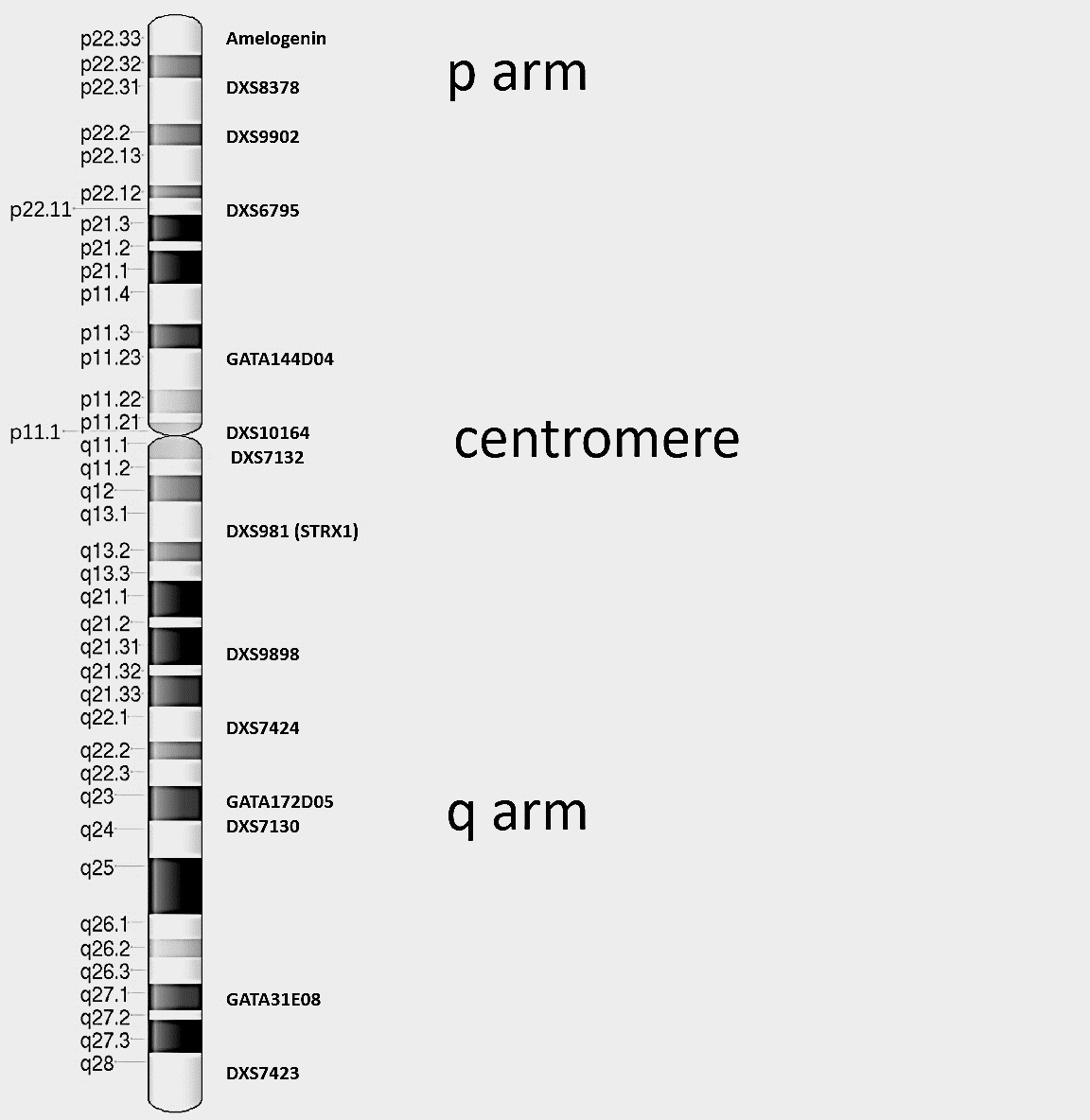


Figure S1

(A)


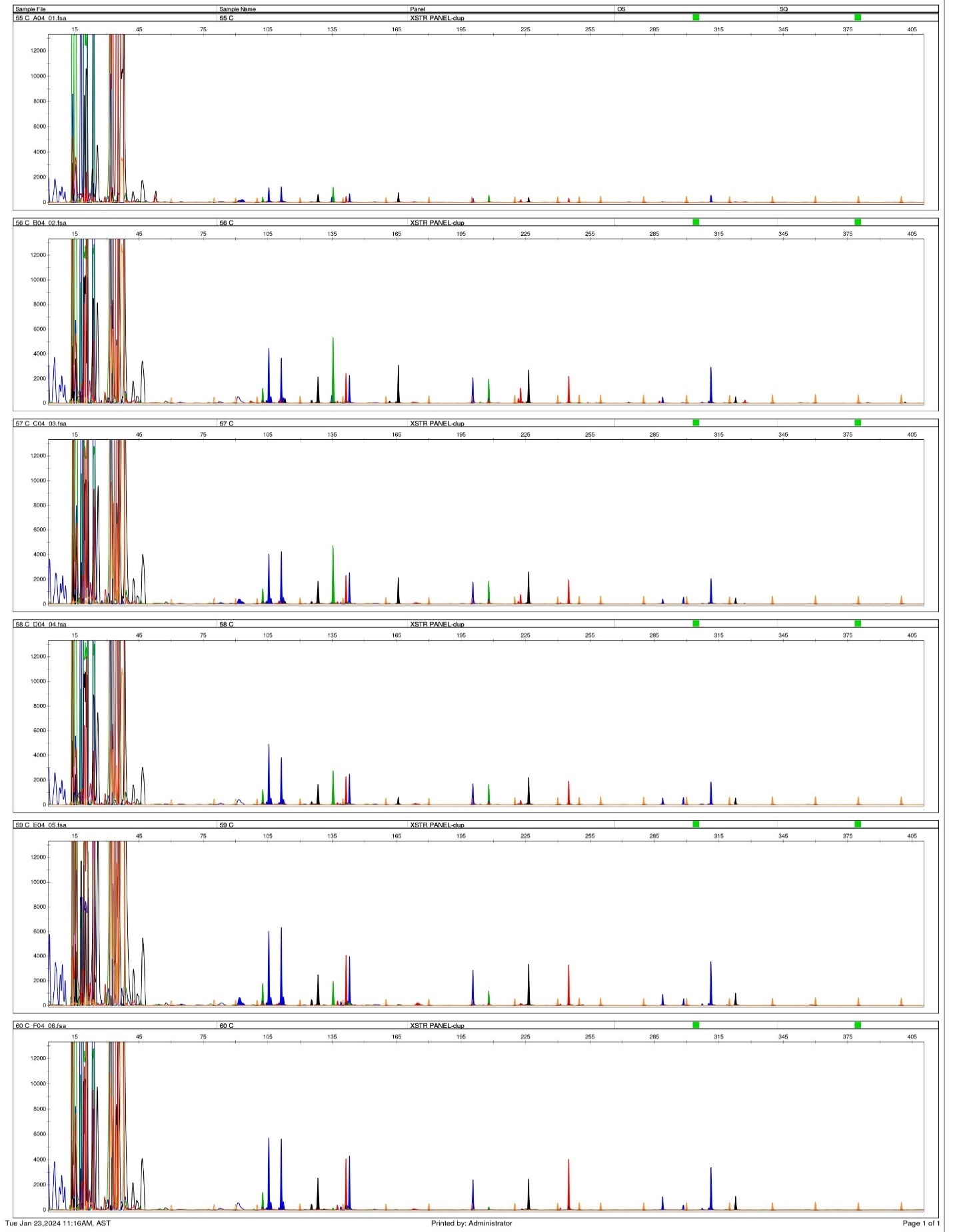


(B)


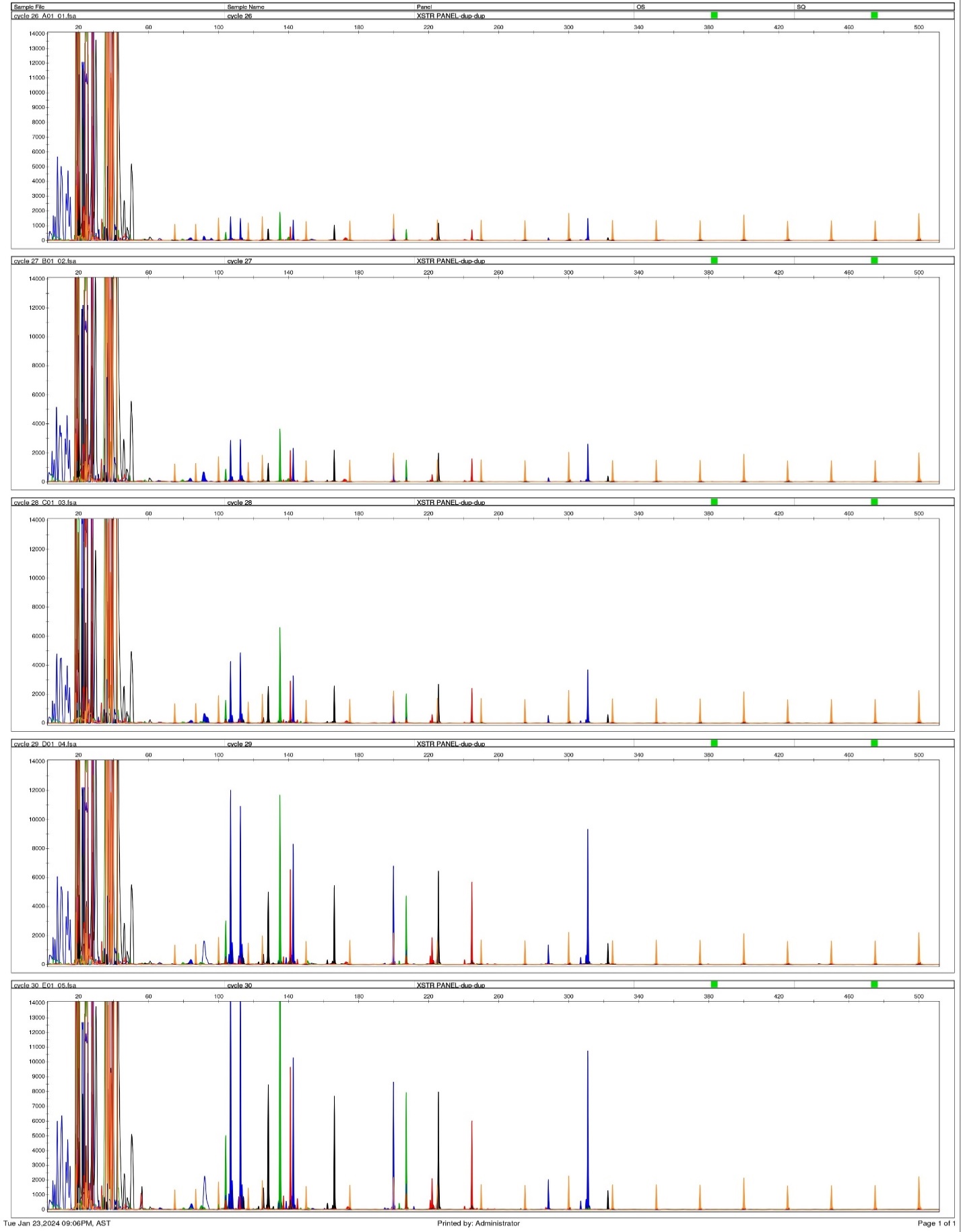


Figure S2


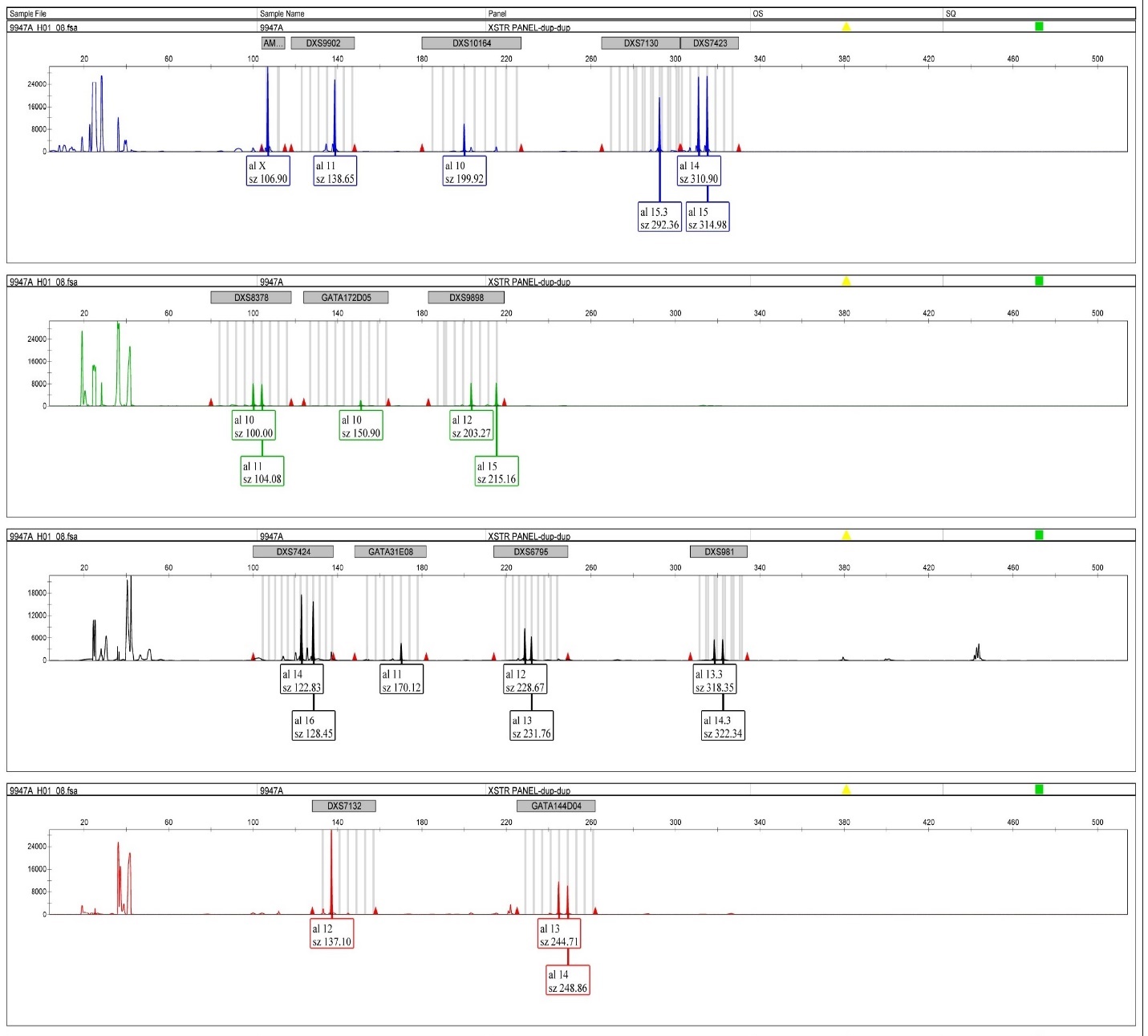


Figure S3


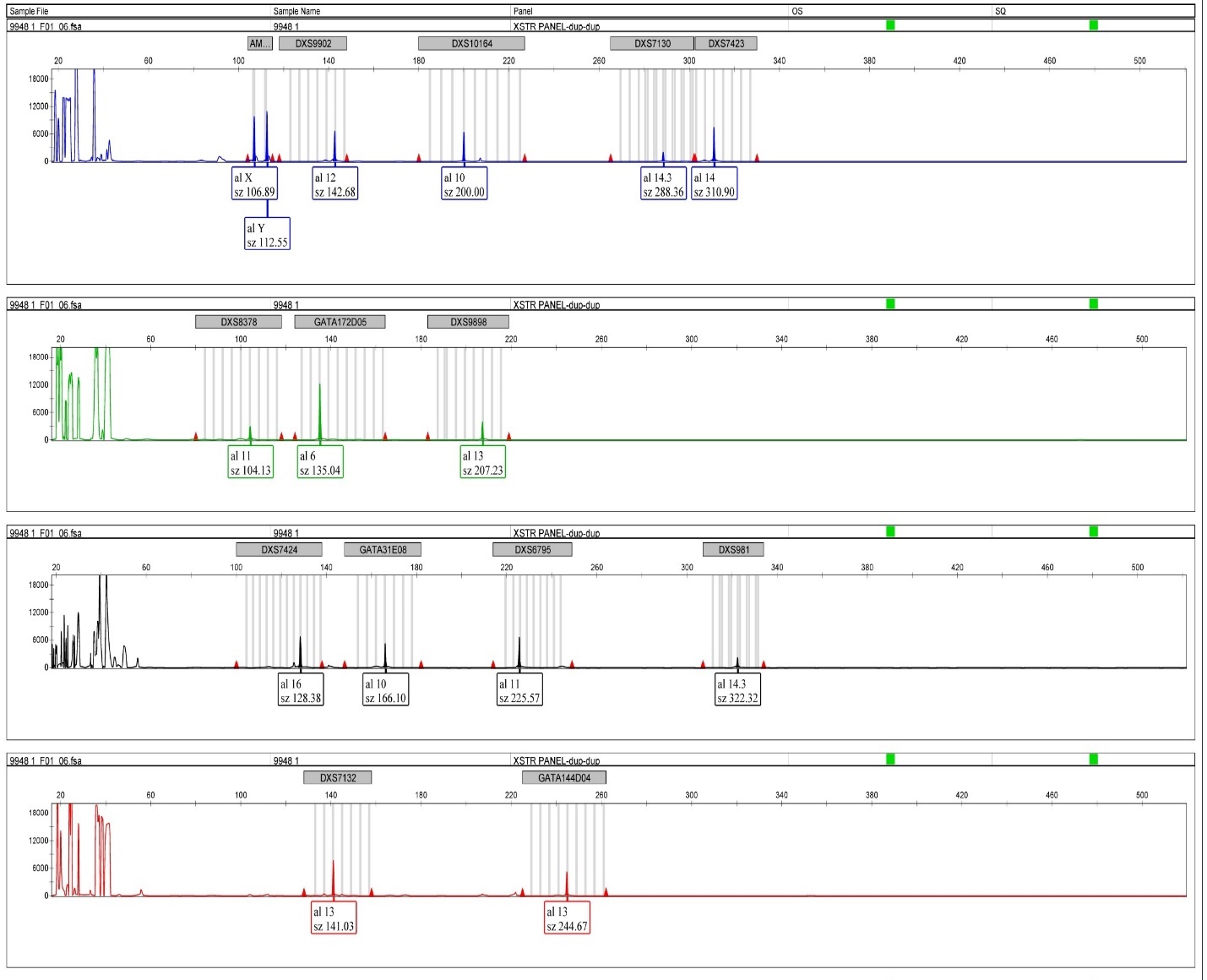


Figure S4


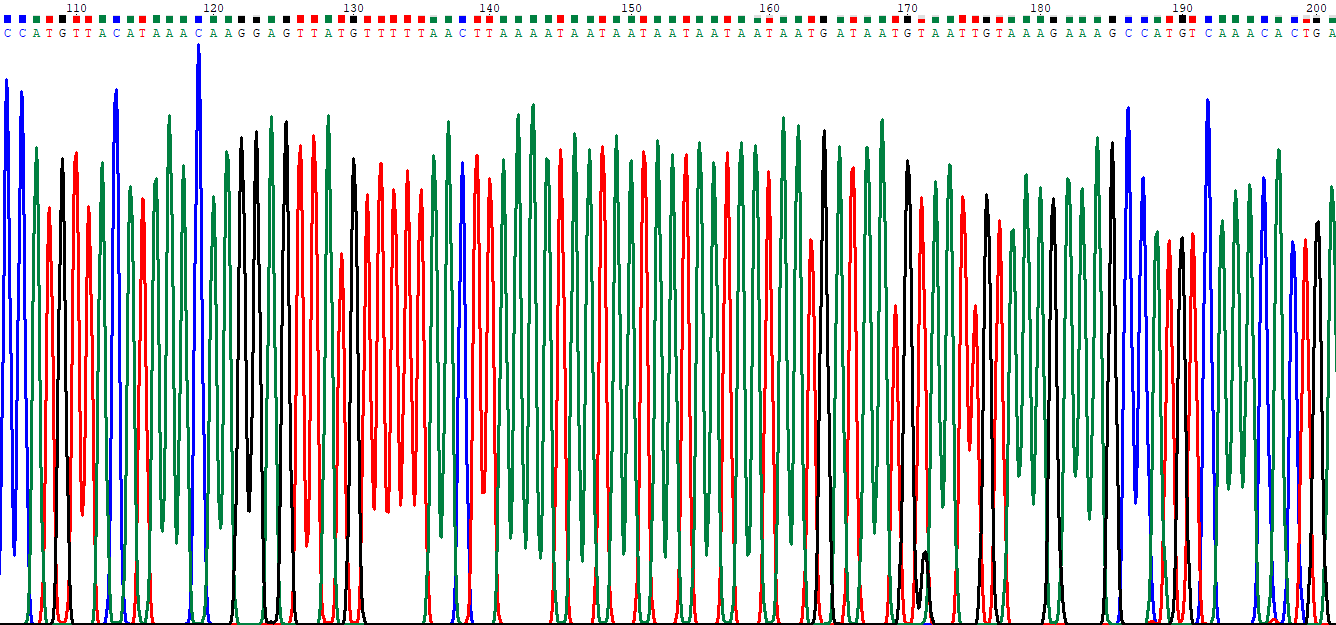


DXS6795 [AAT]7 GAT AAT = 9


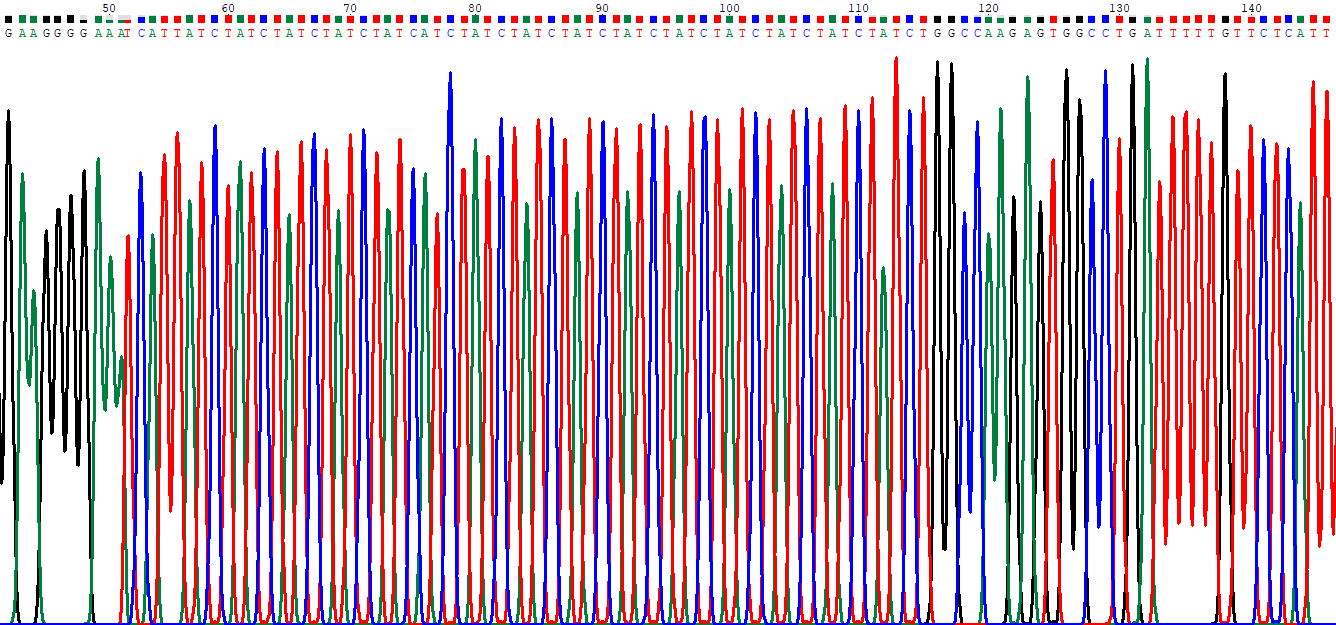


DXS7130 [TATC]5 ATC [TATC]9 = 14.3


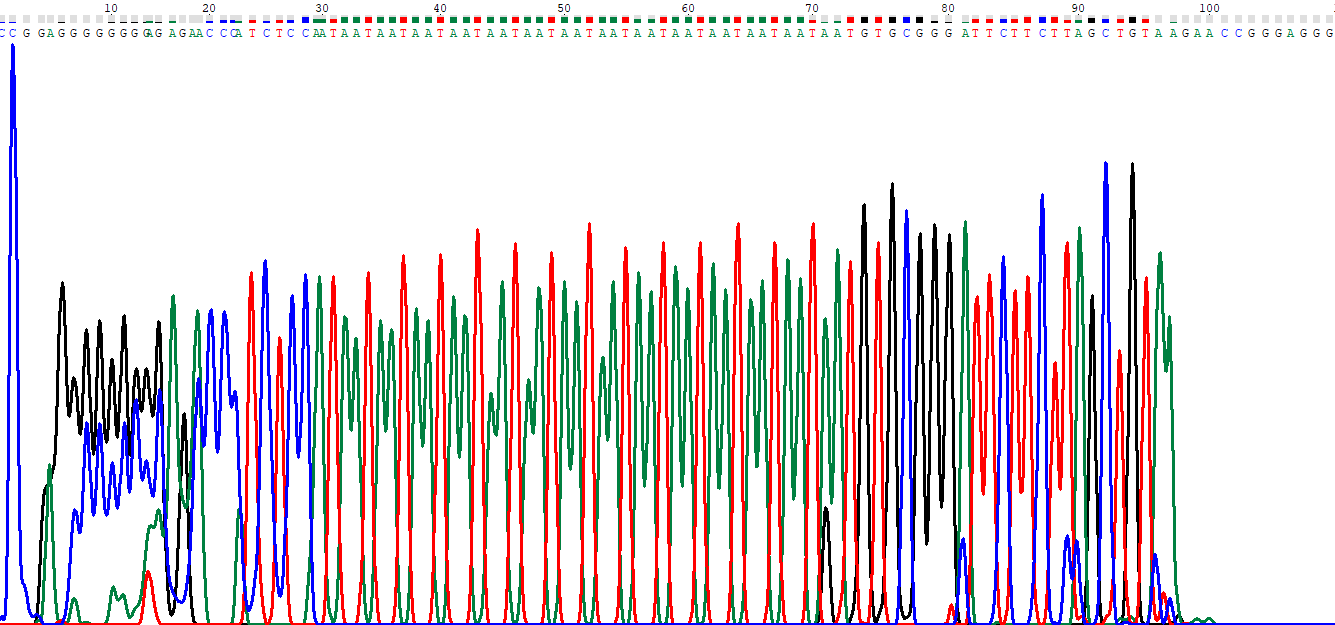


DXS7424 [TAA] = 14


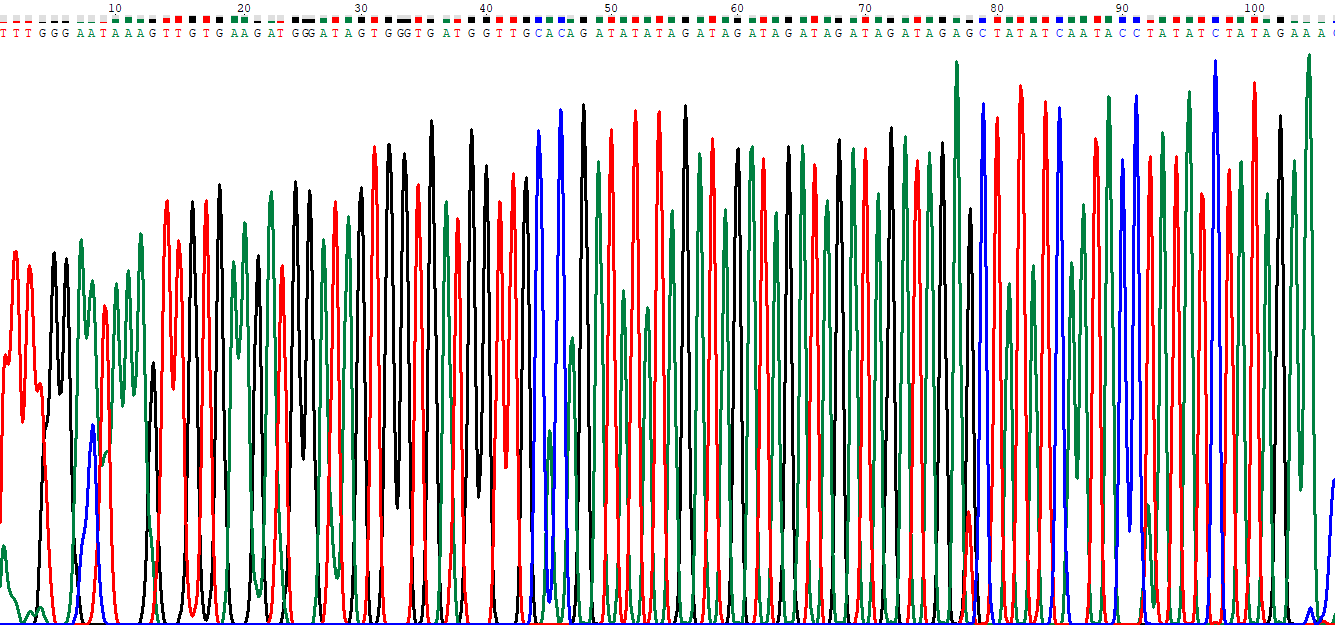


GATA172D05 [TAGA] = 6


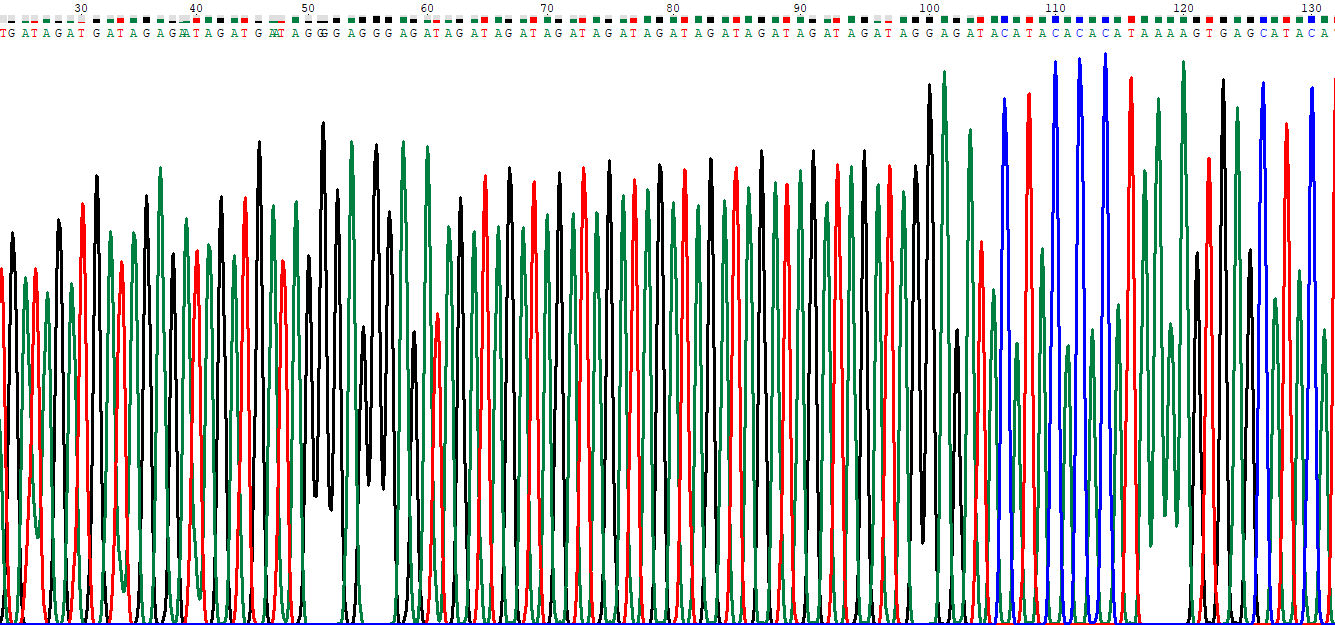


GATA31E08 [AGAT] = 10


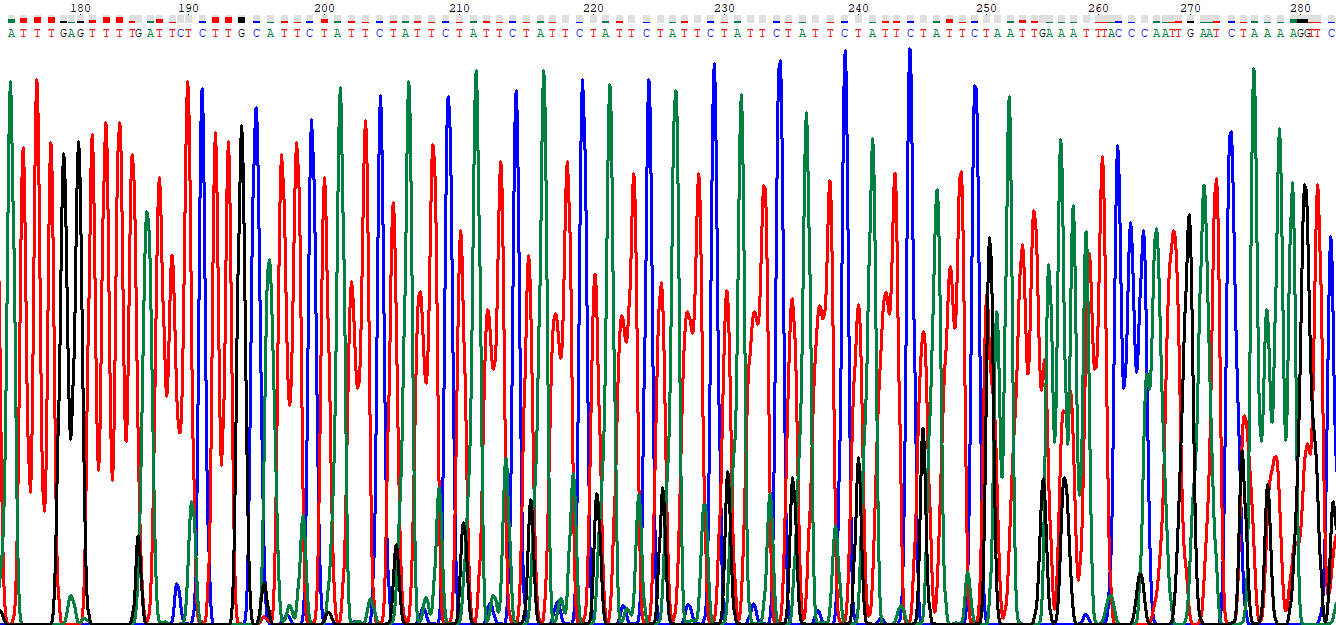


DXS10164 [ATTCT] = 11


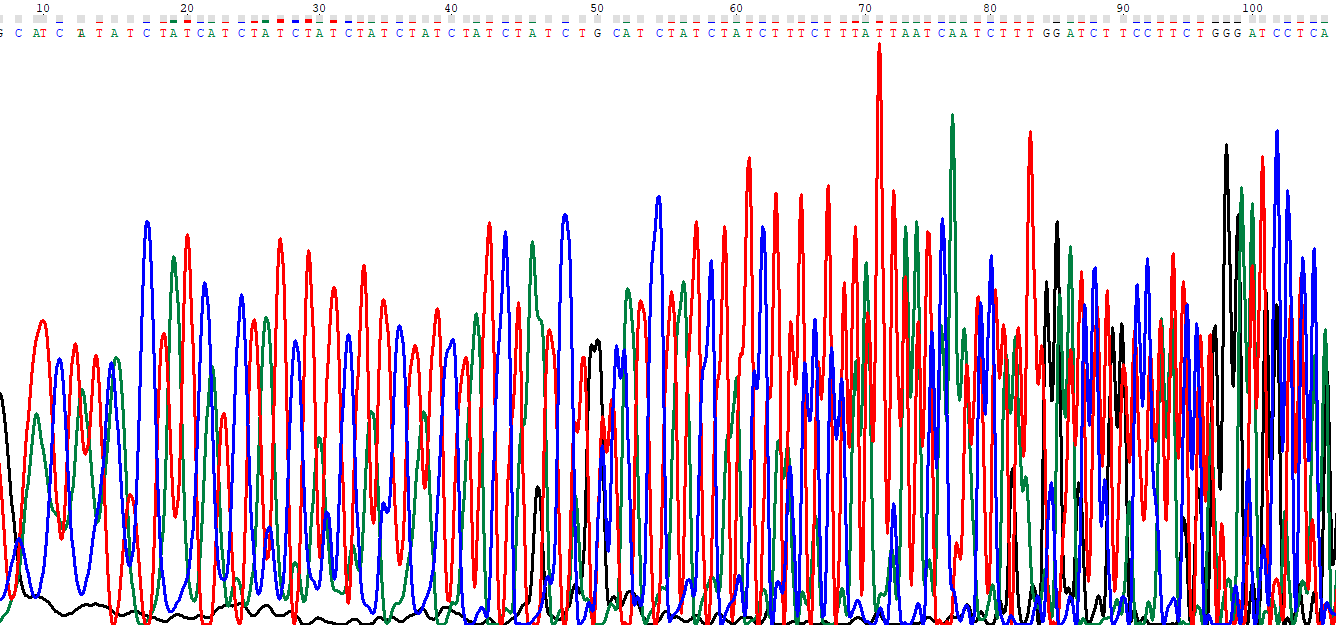


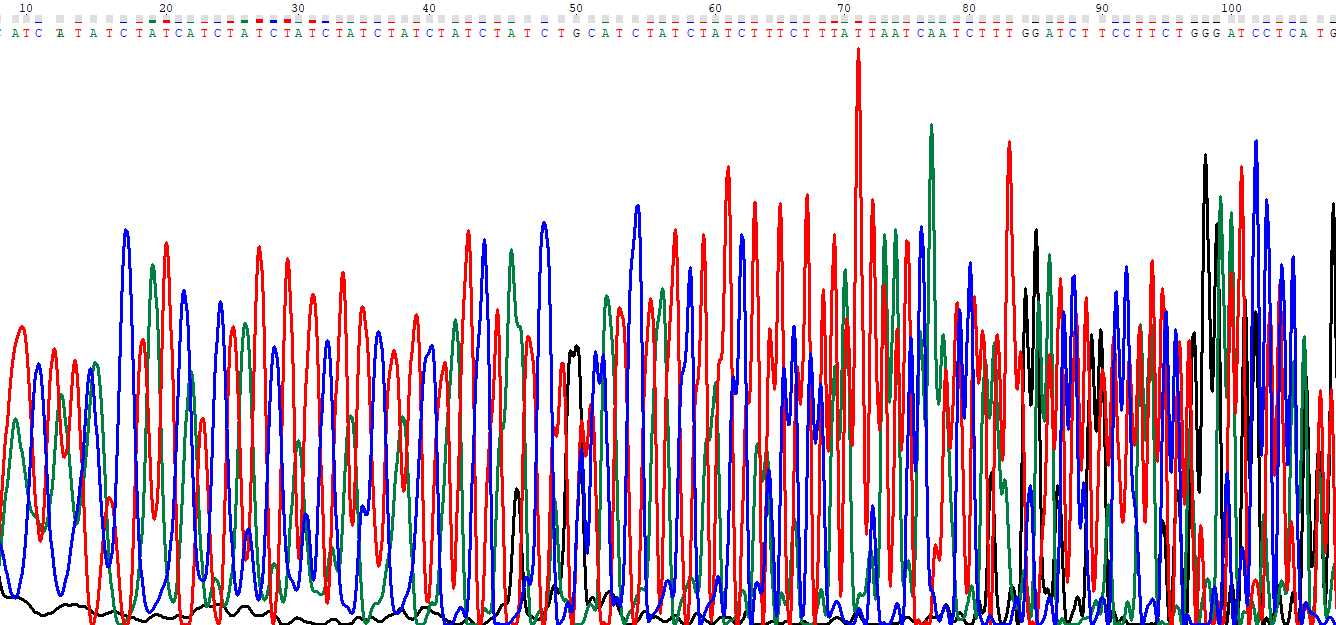


DXS9898 [TATC]2 ATC [TATC]6 = 8.3


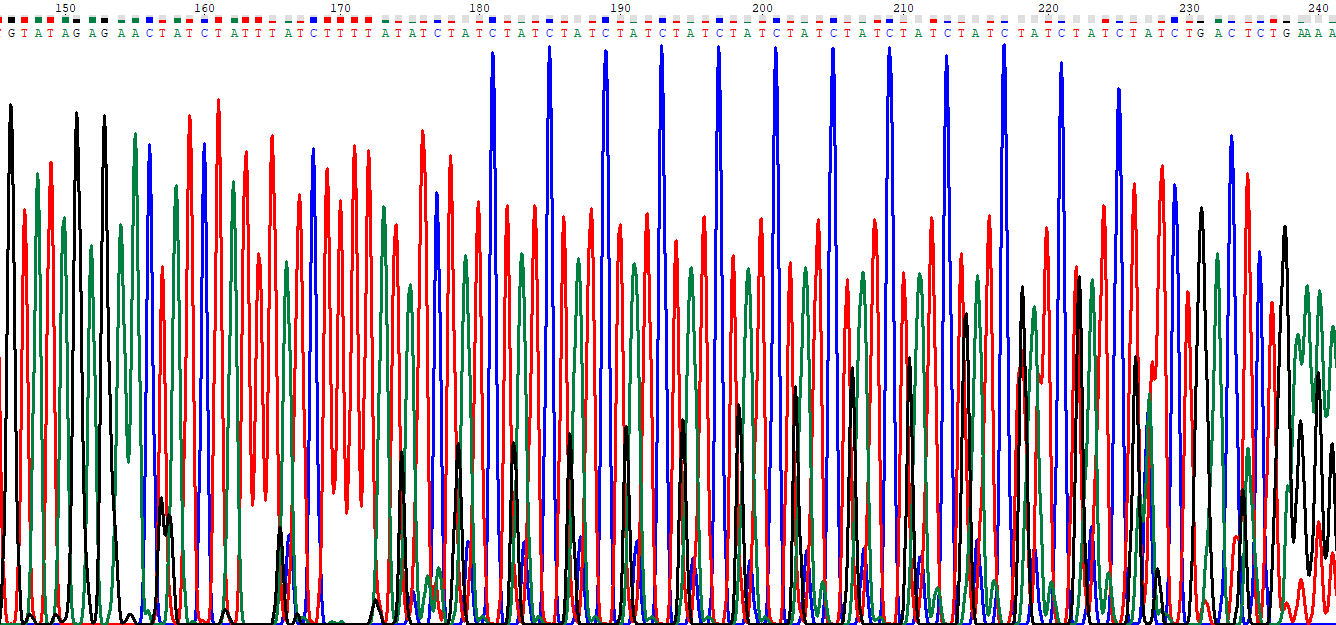


DXS981 [TATC] = 14


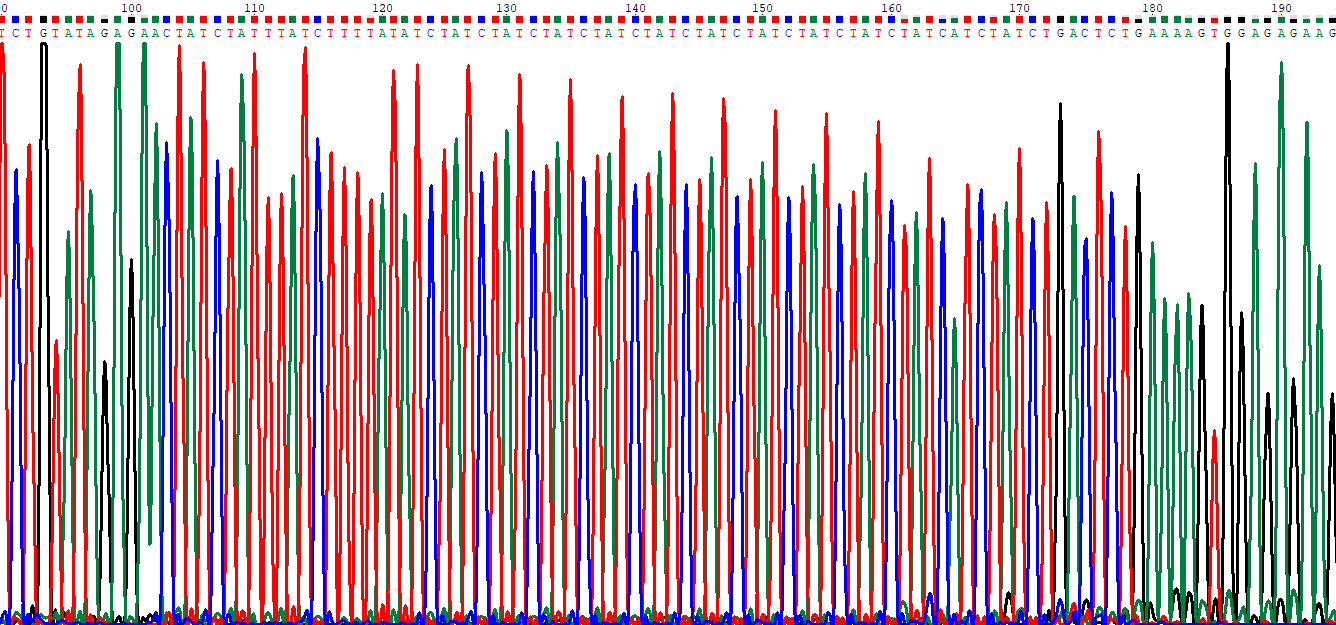


DXS981 [TATC] = 12.3


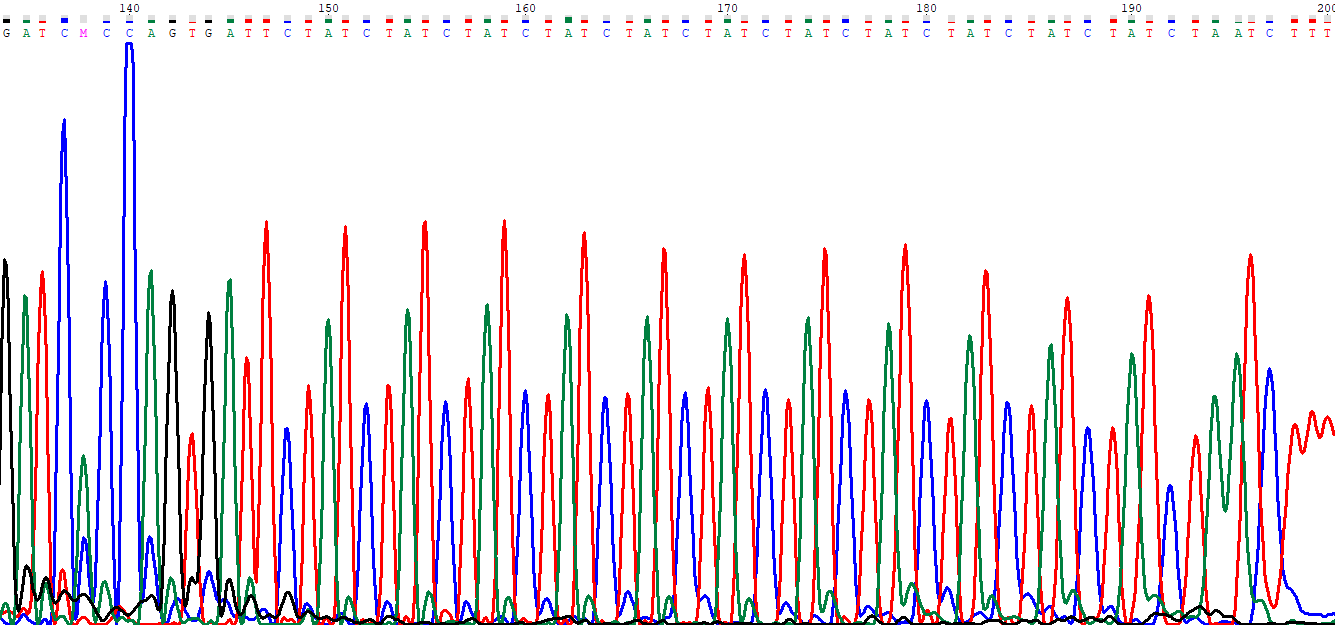


DXS9902 [ATCT] = 11

Figure S5


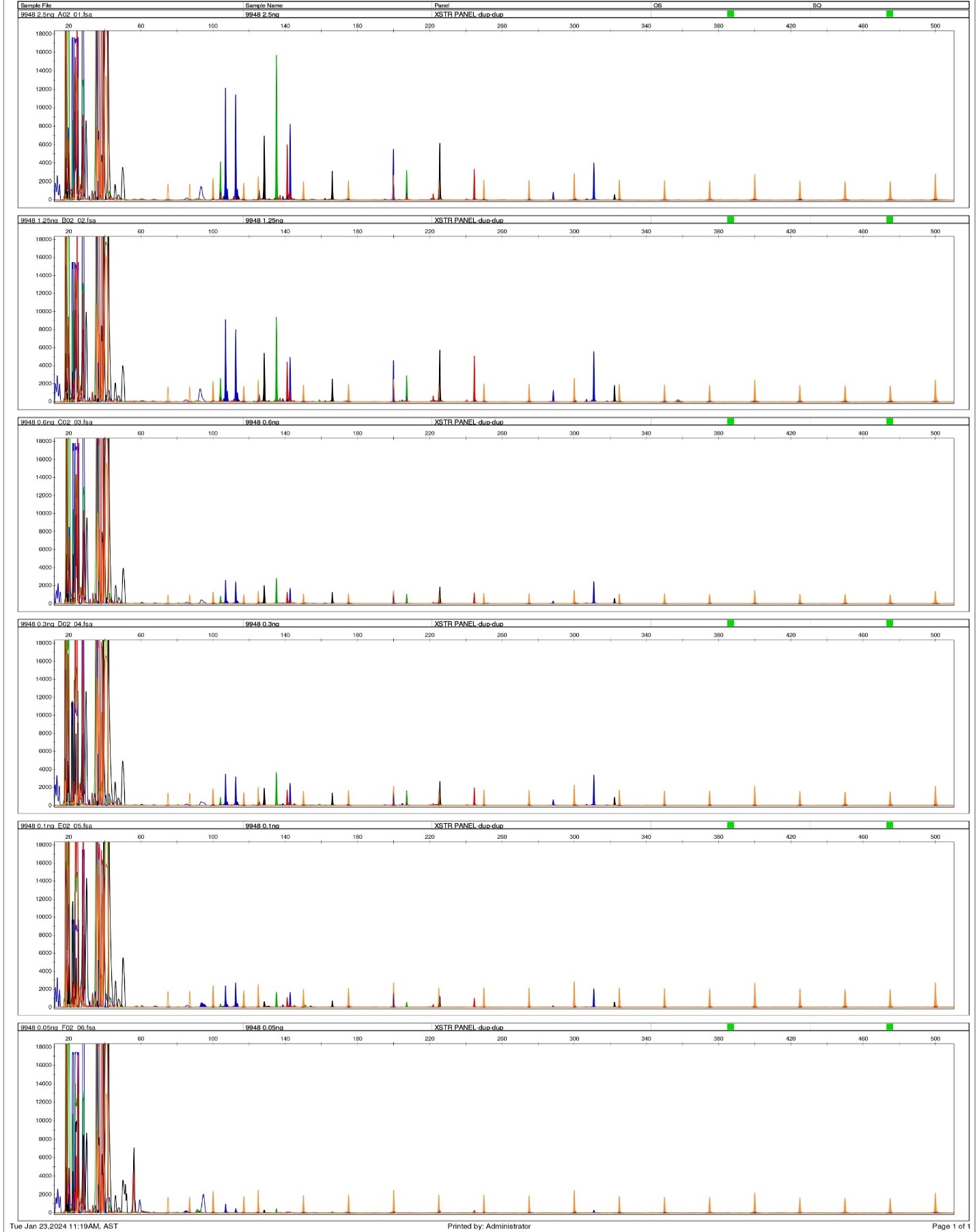


Figure S6


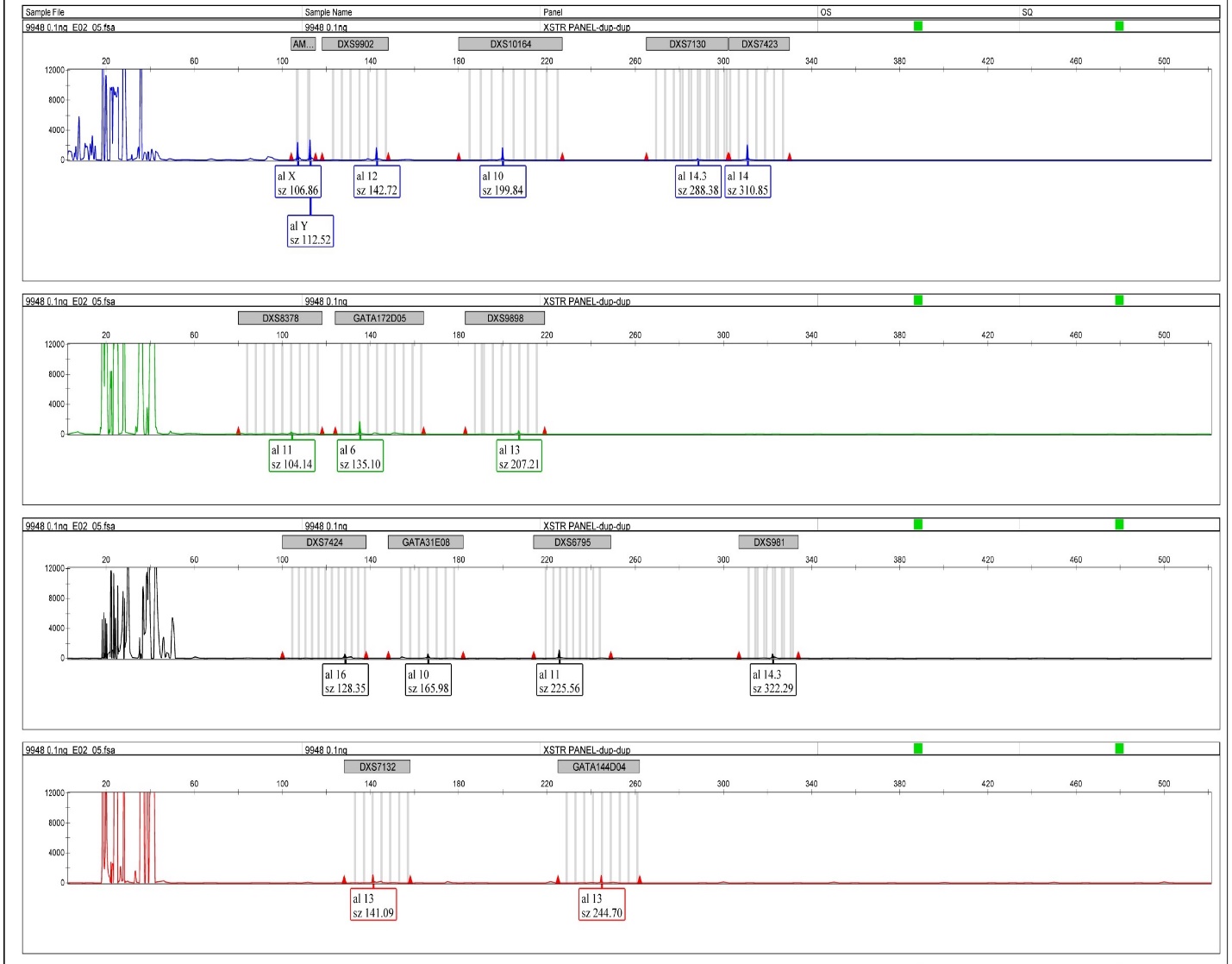


Figure S7

(A)
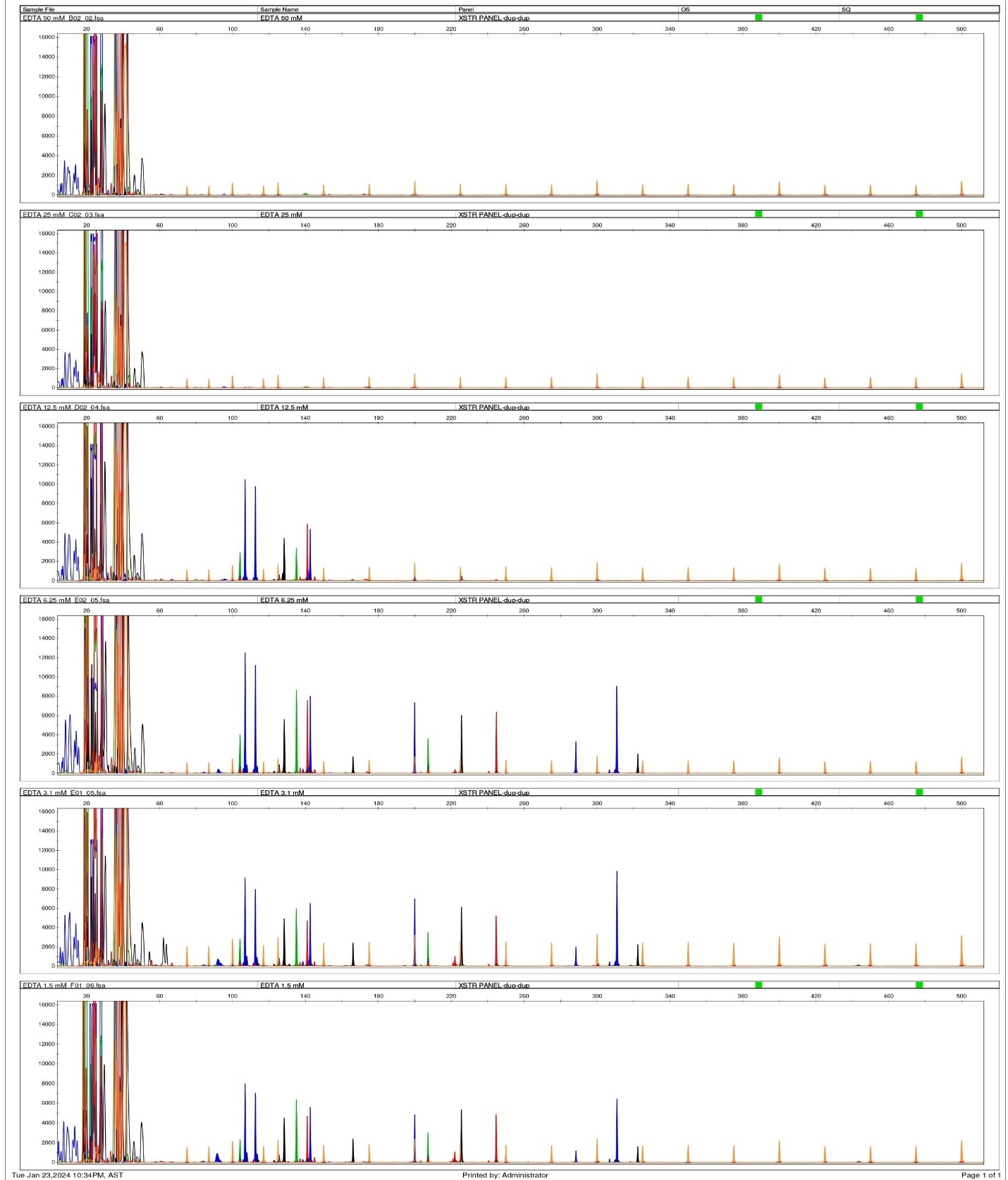


(B)
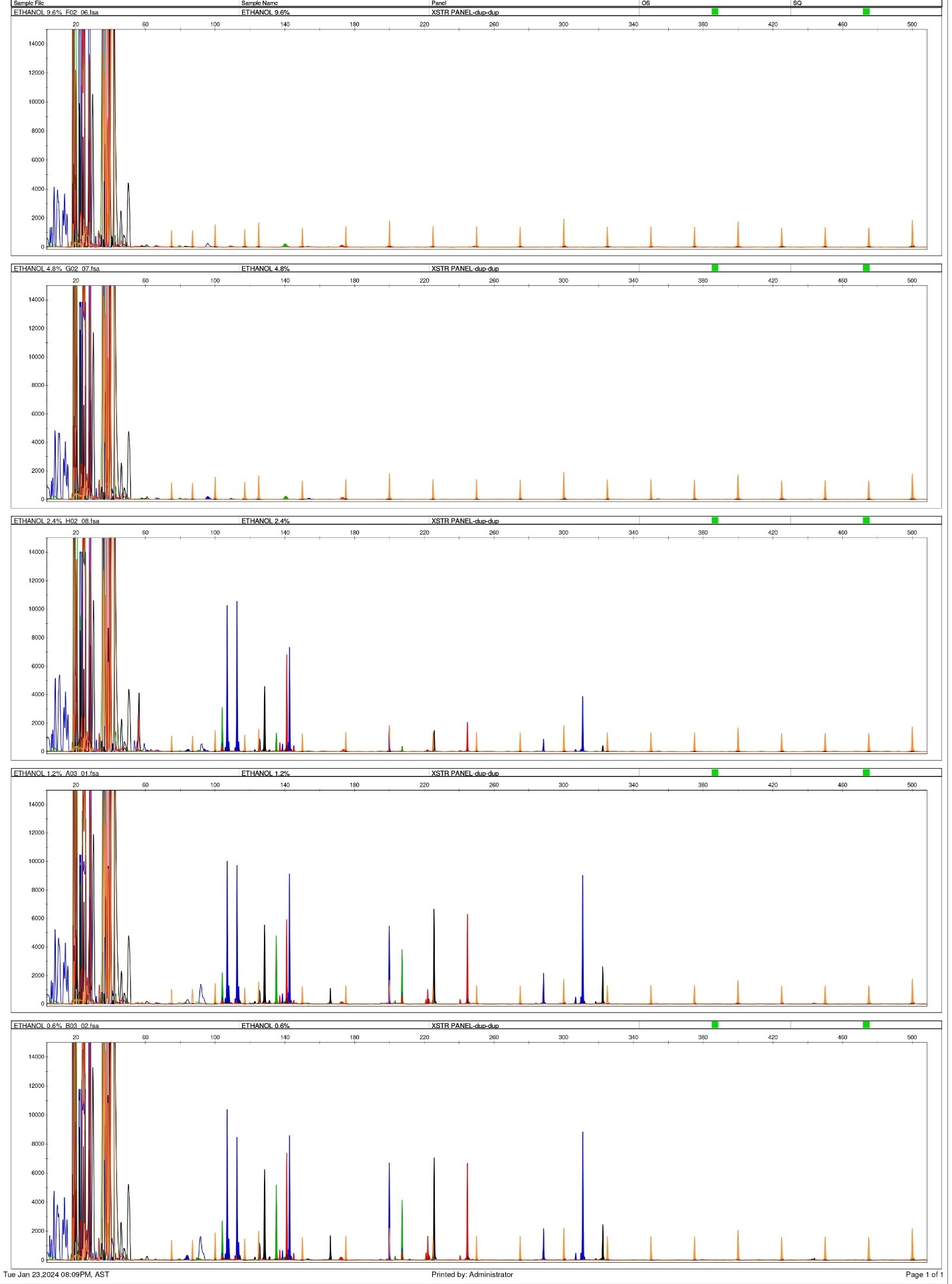


(C)


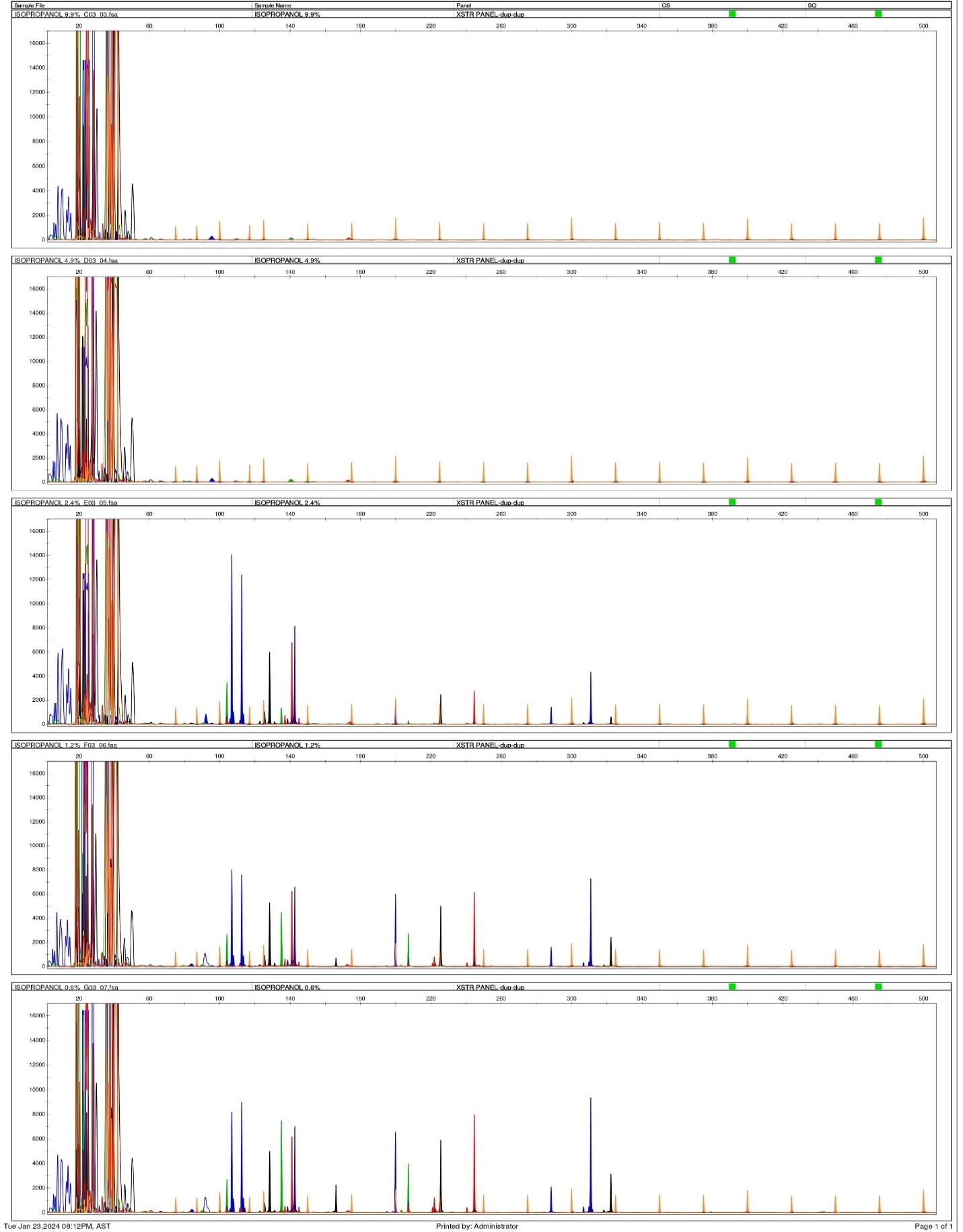


Figure S8


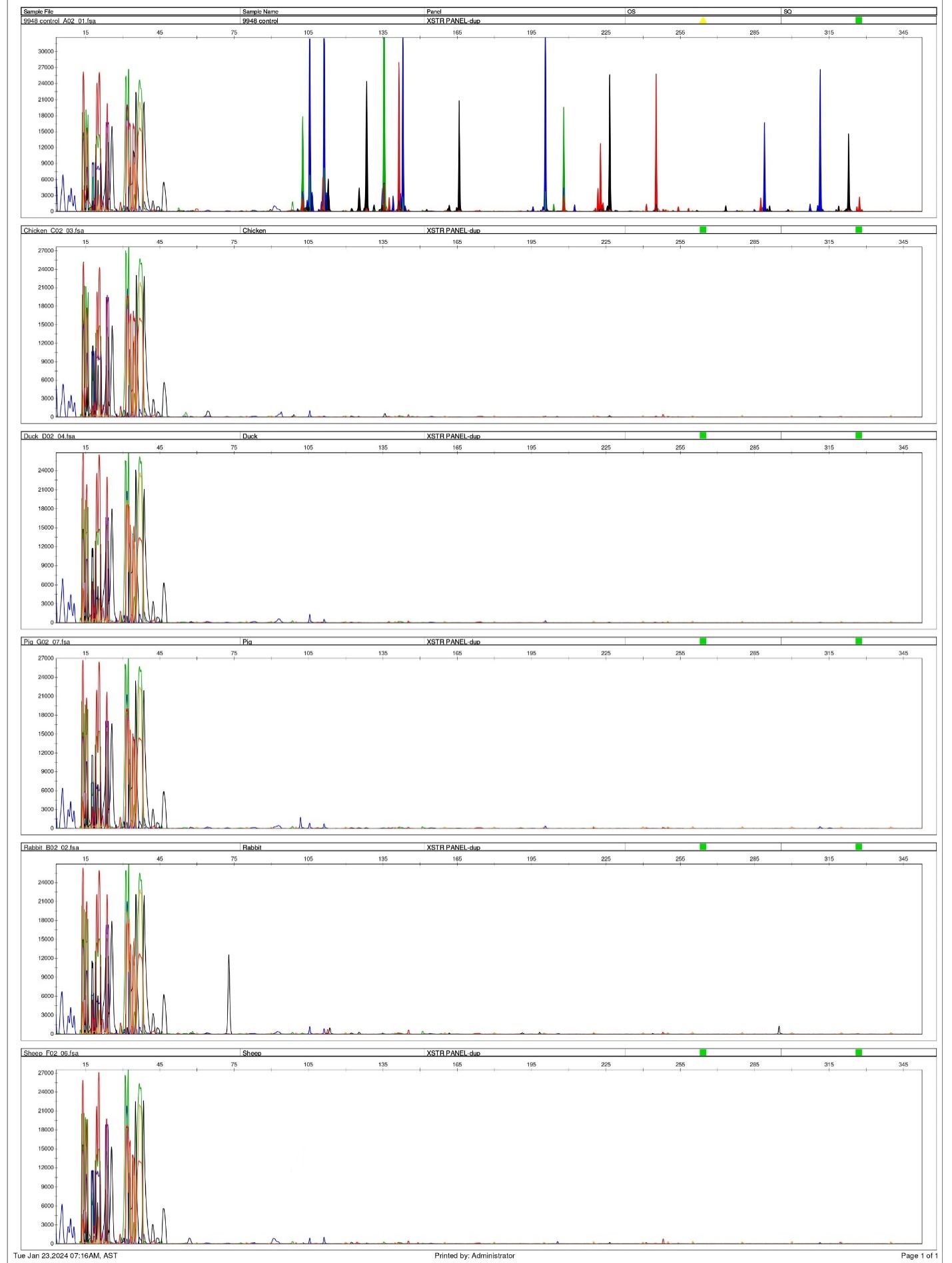


Figure S9

(A)


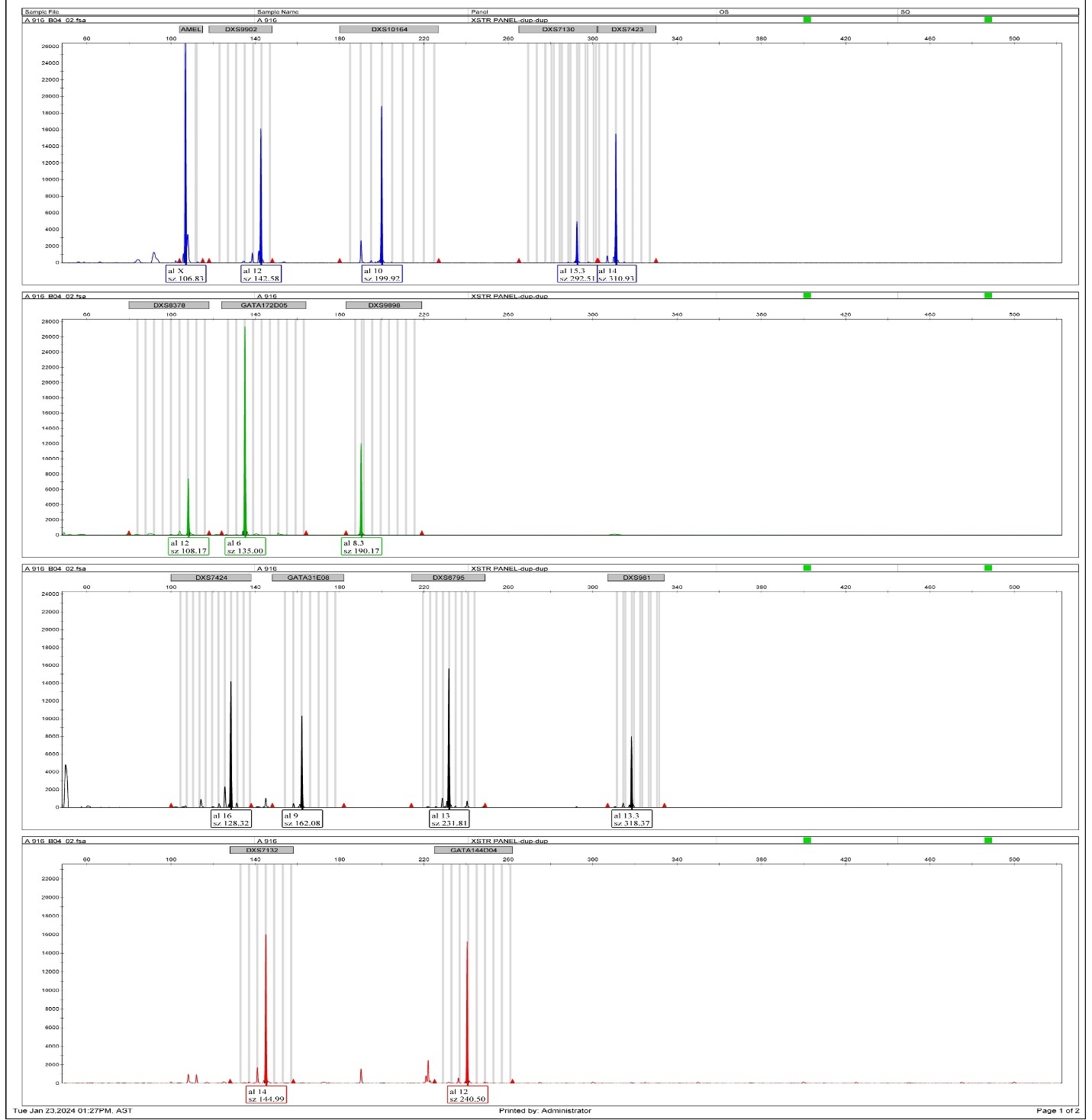


(B)


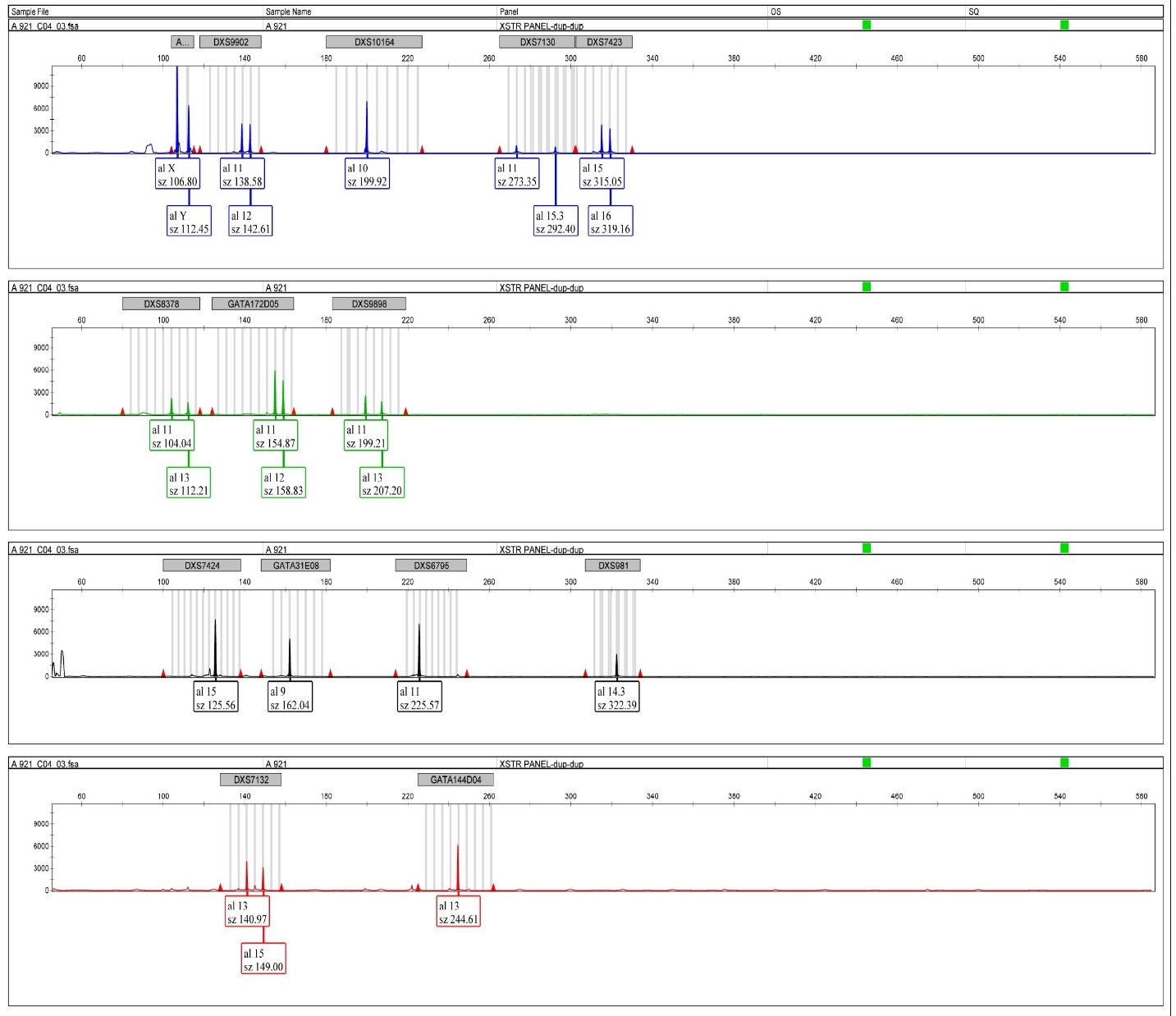


(C)


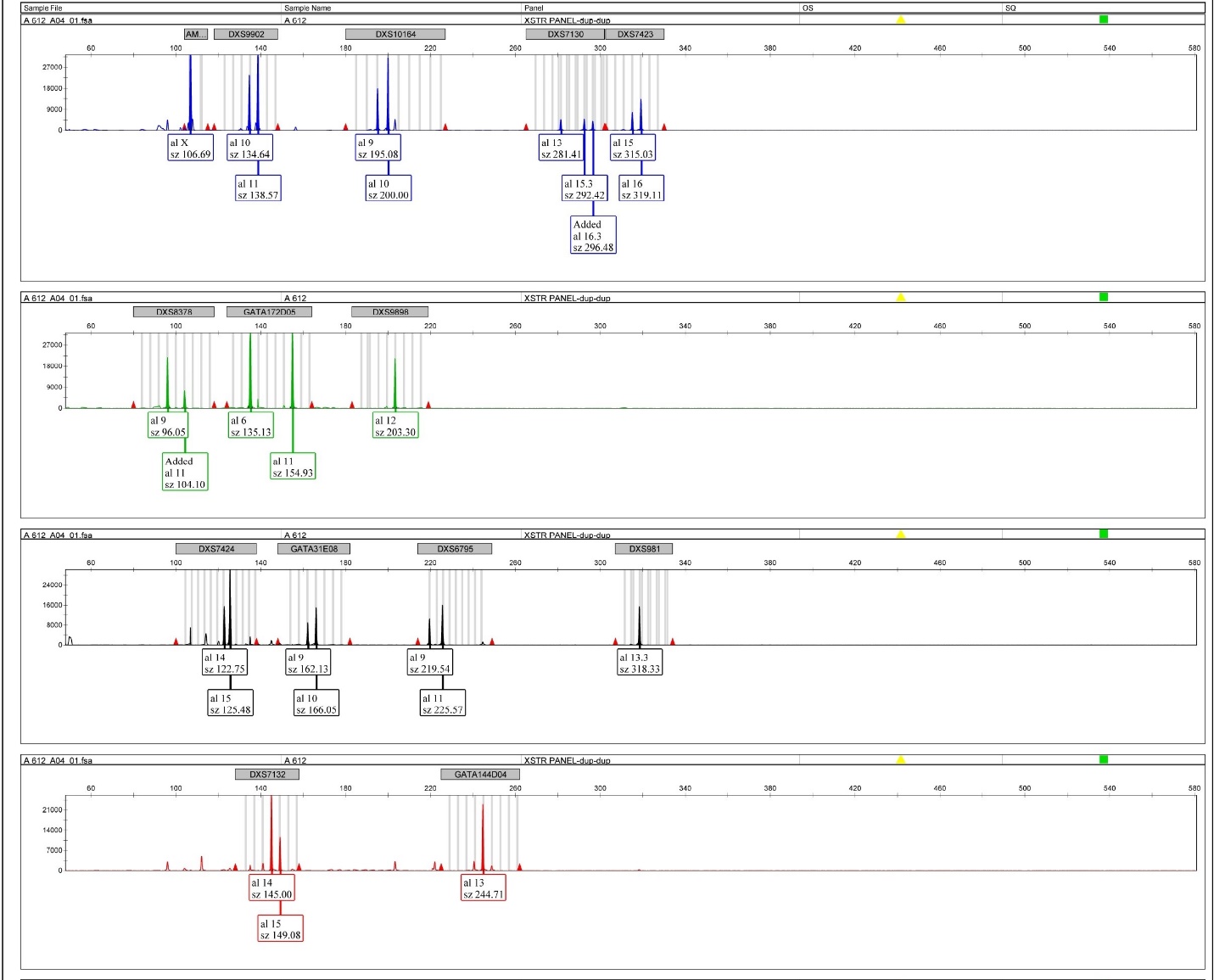


Figure S10

(A)


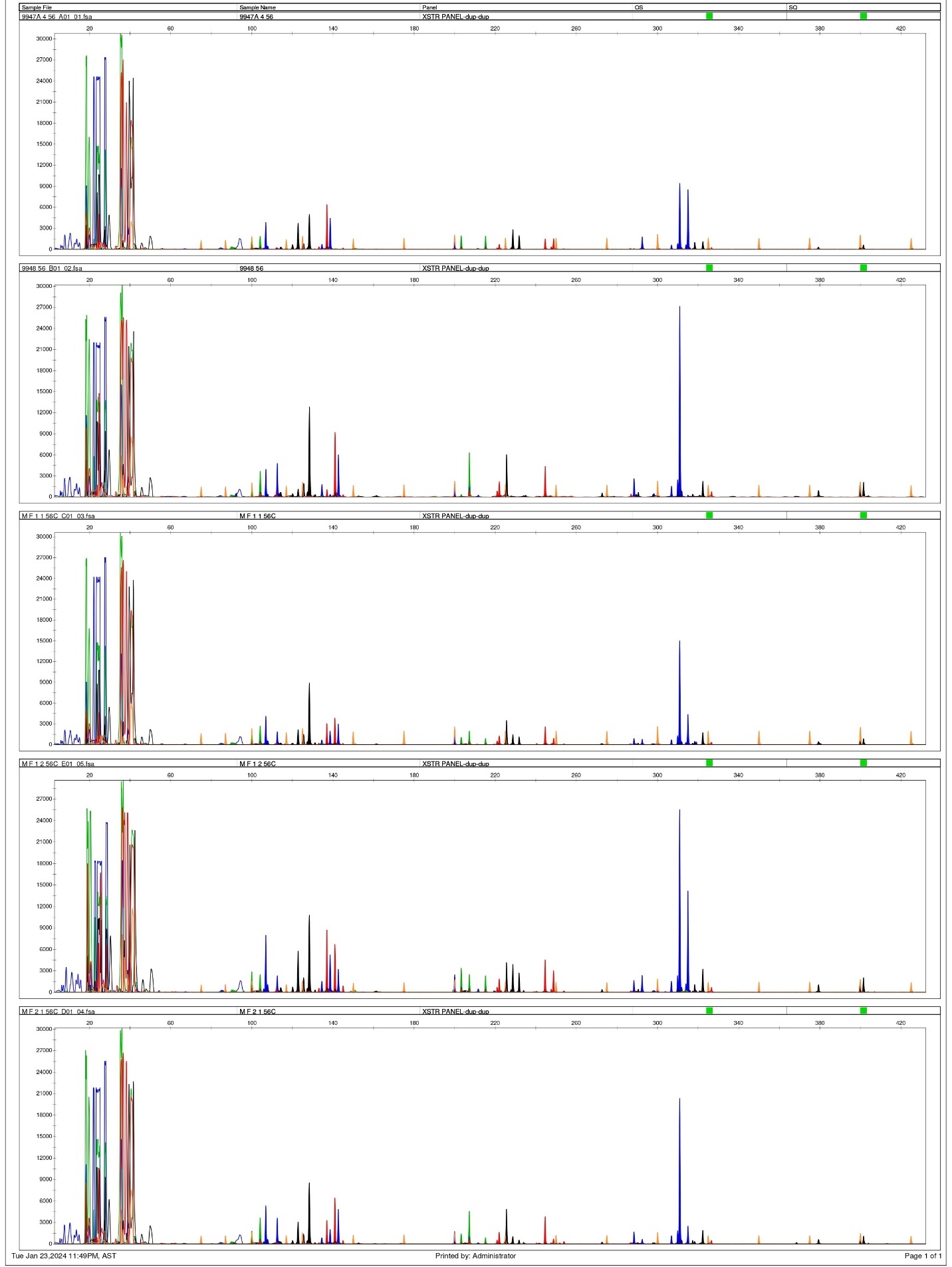


(B)


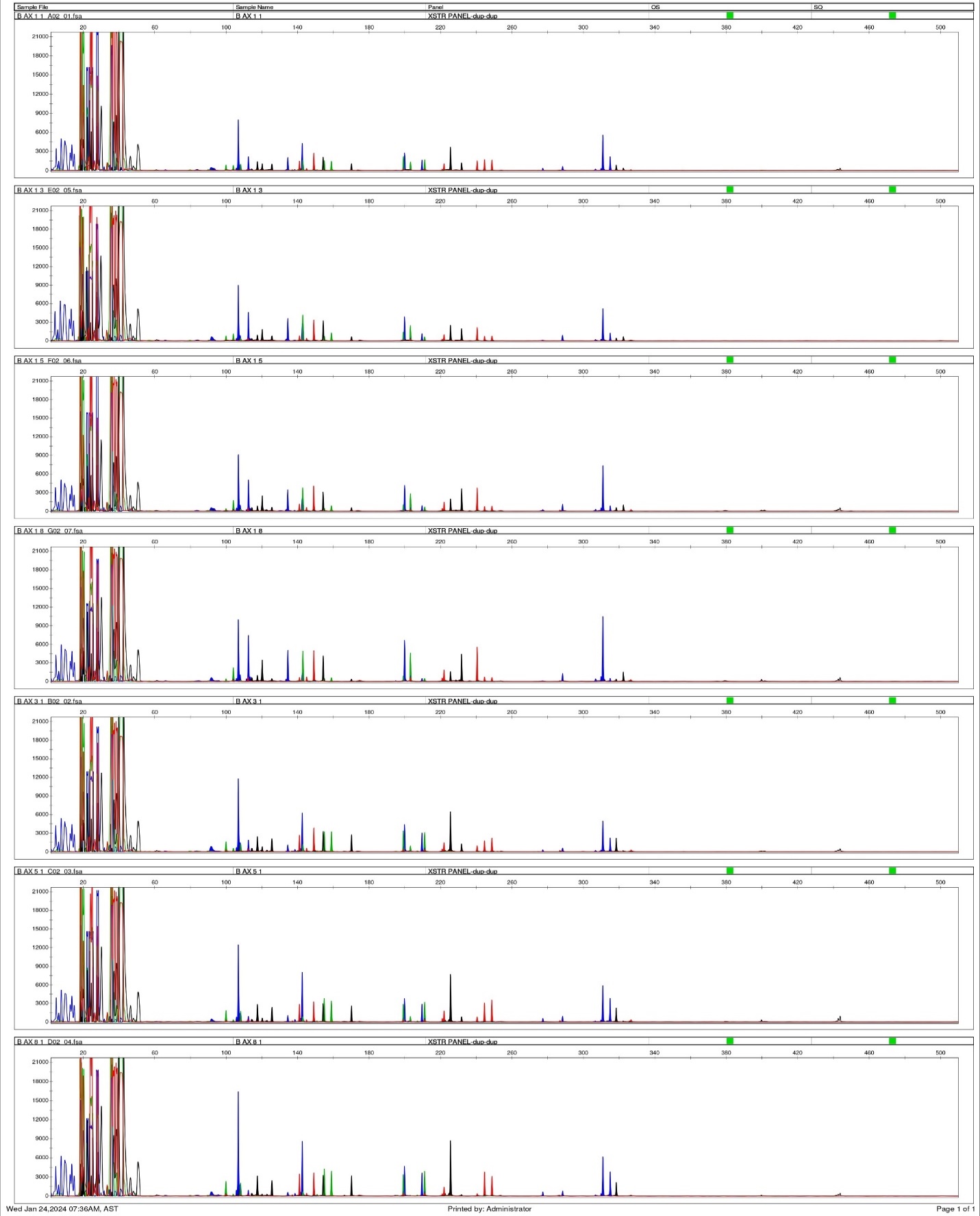


Figure S11
