# Supplementary material for: Genetic Polymorphisms and Forensic Parameters of Thirteen X-Chromosome Markers in the Iraqi Kurdish Population
Source: J Nucleic Acids. 2024 Apr 15;2024:9125094. doi: 10.1155/2024/9125094 (PMC11074882; doi:10.1155/2024/9125094)
Supplement: Supplementary Materials — The following are available online: Figure S1: name and location of the in-house 13 X-STR loci and amelogenin on the X chromosome. Figure S2: evaluation of the PCR conditions for the 13 X-STR multiplex system using (A) gradient PCR with annealing temperatures of 55, 56, 57, 58, 59, and 60°C and (B) number of PCR cycles (26, 27, 28, 29, and 30). All tests were based on 1 ng of 9948 control genomic DNA. Figure S3: electropherogram of the female control DNA 9947A using the 13 X-STR loci. Figure S4: electropherogram of the male genomic control DNA 9948 using the 13 X-STR loci. Figure S5: DNA sequences of 9 amplicons (DXS6795, DXS7130, DXS7424, GATA172D05, GATA31E08, DXS10164, DXS9898, DXS981, and DXS9902) using PCR products of male DNA samples. Figure S6: electropherograms show amplification of the control DNA (9948 male) with the following quantities: 2.5, 1.25, 0.6, 0.3, 0.1, and 0.05 ng to test the sensitivity of the 13 X-STR tool. Figure S7: electropherogram shows the complete profile of the control DNA 9948 male using a DNA amount of 1.25 ng. Figure S8: electropherograms show amplification of the 9948 male control DNA template (1 ng) with inhibitory substances at different concentrations: (A) EDTA (5, 2.5, 1.25, 0.625, 0.31, and 0.15 mM), (B) ethanol (9.6, 4.8, 2.4, 1.2, and 0.6%), and (C) isopropanol (9.9, 4.9, 2.4, 1.2, and 0.6%) in a final volume of 20 μl of PCR reaction. Figure S9: electropherograms show amplification of nonhuman DNA samples from common domestic animals (chicken, duck, pig, rabbit, and sheep) to evaluate the species specificity study. Figure S10: electropherograms show amplification of DNA samples with numerical abnormalities on the X chromosome: (A) Turner syndrome (45, X0), (B) Klinefelter syndrome (47, XXY), and (C) triple X syndrome (trisomy X). Figure S11: electropherograms show amplification of female-male DNA mixtures at different ratios: (A) 9947A female and 9948 male control DNA (1 : 1, 2 : 1, and 1 : 2) and (B) extracted DNA female-ma [file 9125094.f1.zip › Supplementary Tables (1).docx]

**Supplementary Tables**

**Supplementary Table S1:** Table shows genetic DNA profiles and the alleles found at each locus of control DNA 9947A female and 9948 male using the in-house 13 X-STR markers.

| **Locus** | **9947A female** | **9948 male** |
| --- | --- | --- |
| DXS9902 | 11,11 | 12 |
| DXS10164 | 10,10 | 10 |
| DXS7130 | 15.3 | 14.3 |
| DXS7423 | 14,15 | 14 |
| DXS8378 | 10,11 | 11 |
| GATA172D05 | 10,10 | 6 |
| DXS9898 | 12,15 | 13 |
| DXS 7424 | 14,16 | 16 |
| GATA31E08 | 11,11 | 10 |
| DXS6795 | 12,13 | 11 |
| DXS981 | 13.3, 14.3 | 14.3 |
| DXS7132 | 12,12 | 13 |
| GATA 144 D04 | 13,14 | 13 |

**Supplementary Table S2:** Genetic profiles of the 13 X-STR loci were obtained from 159 unrelated Kurd males.

| **Sample name** | **DXS9902** | **DXS10164** | **DXS7130** | **DXS7423** | **DXS8378** | **GATA172D05** | **DXS9898** | **DXS7424** | **GATA31E08** | **DXS6795** | **DXS981** | **DXS7132** | **GATA144D04** |
| --- | --- | --- | --- | --- | --- | --- | --- | --- | --- | --- | --- | --- | --- |
| K1 | 12 | 11 | 15.3 | 15 | 10 | 12 | 8.3 | 16 | 10 | 9 | 12.3 | 14 | 13 |
| K2 | 10 | 12 | 14.3 | 14 | 6 | 6 | 8.3 | 15 | 9 | 11 | 13.3 | 14 | 10 |
| K3 | 10 | 10 | 15.3 | 15 | 12 | 10 | 8.3 | 12 | 9 | 11 | 13.3 | 14 | 13 |
| K4 | 12 | 10 | 15.3 | 14 | 11 | 9 | 11 | 16 | 9 | 10 | 12.3 | 14 | 13 |
| K5 | 12 | 8 | 15.3 | 14 | 11 | 5 | 8.3 | 14 | 9 | 10 | 12.3 | 13 | 14 |
| K6 | 11 | 12 | 15.3 | 16 | 12 | 10 | 12 | 17 | 11 | 13 | 13.3 | 15 | 14 |
| K7 | 12 | 10 | 15.3 | 17 | 12 | 6 | 12 | 15 | 9 | 9 | 13.3 | 14 | 14 |
| K8 | 12 | 10 | 15.3 | 15 | 11 | 6 | 11 | 16 | 10 | 13 | 13.3 | 14 | 13 |
| K9 | 11 | 12 | 15.3 | 15 | 11 | 9 | 12 | 15 | 10 | 13 | 15.3 | 14 | 14 |
| K10 | 11 | 10 | 14.3 | 15 | 12 | 9 | 12 | 11 | 7 | 11 | 13.3 | 14 | 15 |
| K11 | 11 | 11 | 13 | 14 | 12 | 6 | 8.3 | 10 | 11 | 13 | 13.3 | 12 | 13 |
| K12 | 11 | 10 | 16.3 | 15 | 11 | 12 | 8.3 | 16 | 10 | 14 | 13.3 | 13 | 12 |
| K13 | 12 | 9 | 14.3 | 17 | 11 | 10 | 11 | 16 | 12 | 9 | 13.3 | 15 | 13 |
| K14 | 12 | 10 | 15.3 | 14 | 9 | 10 | 13 | 14 | 10 | 9 | 12.3 | 12 | 13 |
| K15 | 10 | 11 | 13.3 | 14 | 10 | 8 | 12 | 16 | 7 | 13 | 13.3 | 13 | 10 |
| K16 | 10 | 11 | 15.3 | 17 | 10 | 8 | 12 | 14 | 11 | 13 | 13.3 | 13 | 14 |
| K17 | 11 | 10 | 15.3 | 14 | 11 | 10 | 11 | 10 | 7 | 11 | 13.3 | 13 | 12 |
| K18 | 11 | 11 | 14.3 | 16 | 10 | 6 | 8.3 | 14 | 10 | 9 | 14.3 | 14 | 14 |
| K19 | 11 | 11 | 17.3 | 16 | 10 | 8 | 12 | 16 | 9 | 11 | 14.3 | 13 | 15 |
| K20 | 10 | 10 | 11 | 15 | 11 | 8 | 8.3 | 15 | 7 | 11 | 14.3 | 16 | 14 |
| K21 | 10 | 10 | 15.3 | 17 | 12 | 9 | 13 | 16 | 7 | 13 | 14.3 | 13 | 13 |
| K22 | 12 | 12 | 15.3 | 15 | 10 | 10 | 12 | 15 | 7 | 11 | 14.3 | 14 | 13 |
| K23 | 10 | 10 | 14.3 | 14 | 10 | 8 | 8.3 | 17 | 7 | 9 | 13.3 | 15 | 12 |
| K24 | 10 | 11 | 15.3 | 16 | 11 | 6 | 13 | 13 | 9 | 9 | 14.3 | 12 | 12 |
| K25 | 9 | 10 | 14.3 | 17 | 6 | 10 | 12 | 15 | 10 | 13 | 13.3 | 13 | 13 |
| K26 | 10 | 11 | 15.3 | 14 | 10 | 6 | 8.3 | 14 | 7 | 9 | 13.3 | 14 | 13 |
| K27 | 11 | 10 | 15.3 | 16 | 12 | 11 | 11 | 15 | 7 | 11 | 15.3 | 12 | 13 |
| K28 | 12 | 11 | 14.3 | 14 | 11 | 9 | 12 | 15 | 12 | 10 | 13.3 | 15 | 13 |
| K29 | 11 | 10 | 14.3 | 14 | 11 | 6 | 12 | 16 | 10 | 9 | 13.3 | 13 | 13 |
| K30 | 10 | 10 | 15.3 | 14 | 10 | 10 | 8.3 | 16 | 10 | 9 | 14.3 | 14 | 16 |
| K31 | 11 | 11 | 15.3 | 15 | 12 | 8 | 12 | 16 | 9 | 11 | 14.3 | 14 | 12 |
| K32 | 11 | 12 | 15.3 | 15 | 11 | 10 | 8.3 | 15 | 12 | 9 | 13.3 | 12 | 14 |
| K33 | 10 | 11 | 16.3 | 15 | 10 | 11 | 13 | 13 | 9 | 11 | 13.3 | 15 | 15 |
| K34 | 12 | 11 | 15.3 | 17 | 12 | 6 | 8.3 | 10 | 11 | 11 | 14.3 | 15 | 14 |
| K35 | 11 | 11 | 15.3 | 14 | 12 | 11 | 8.3 | 15 | 7 | 11 | 13.3 | 14 | 15 |
| K36 | 12 | 10 | 14.3 | 15 | 11 | 12 | 12 | 12 | 9 | 11 | 13.3 | 15 | 13 |
| K37 | 10 | 10 | 12 | 15 | 12 | 8 | 11 | 16 | 7 | 11 | 13.3 | 14 | 13 |
| K38 | 11 | 11 | 12 | 15 | 12 | 12 | 11 | 13 | 9 | 9 | 13.3 | 14 | 13 |
| K39 | 10 | 10 | 14.3 | 15 | 12 | 9 | 12 | 14 | 9 | 11 | 13.3 | 13 | 13 |
| K40 | 10 | 15 | 15.3 | 15 | 12 | 10 | 11 | 15 | 9 | 11 | 13.3 | 15 | 13 |
| K41 | 10 | 10 | 14.3 | 17 | 11 | 9 | 12 | 14 | 9 | 9 | 14.3 | 13 | 14 |
| K42 | 10 | 12 | 16.3 | 14 | 10 | 6 | 10 | 15 | 9 | 9 | 15.3 | 13 | 13 |
| K43 | 11 | 10 | 15.3 | 14 | 12 | 11 | 11 | 13 | 10 | 11 | 16.3 | 13 | 15 |
| K44 | 12 | 11 | 15.3 | 13 | 12 | 8 | 12 | 16 | 12 | 11 | 12.3 | 13 | 15 |
| K45 | 10 | 11 | 16.3 | 14 | 11 | 11 | 11 | 15 | 10 | 11 | 13.3 | 14 | 13 |
| K46 | 10 | 12 | 14.3 | 14 | 11 | 6 | 8.3 | 13 | 11 | 9 | 12.3 | 13 | 13 |
| K47 | 10 | 10 | 15.3 | 15 | 11 | 6 | 11 | 15 | 11 | 9 | 12.3 | 12 | 13 |
| K48 | 9 | 10 | 14.3 | 14 | 11 | 6 | 11 | 14 | 11 | 13 | 15.3 | 14 | 13 |
| K49 | 12 | 11 | 14.3 | 14 | 11 | 10 | 13 | 14 | 11 | 11 | 14.3 | 14 | 13 |
| K50 | 10 | 10 | 15.3 | 15 | 12 | 12 | 13 | 15 | 11 | 11 | 13.3 | 14 | 15 |
| K51 | 12 | 11 | 15.3 | 16 | 10 | 8 | 13 | 13 | 11 | 11 | 14.3 | 13 | 11 |
| K52 | 11 | 10 | 15.3 | 15 | 10 | 8 | 8.3 | 15 | 11 | 9 | 12.3 | 15 | 12 |
| K53 | 12 | 11 | 15.3 | 14 | 11 | 6 | 12 | 16 | 11 | 13 | 13.3 | 15 | 13 |
| K54 | 10 | 12 | 15.3 | 14 | 12 | 10 | 11 | 12 | 11 | 11 | 12.3 | 14 | 12 |
| K55 | 10 | 10 | 14.3 | 16 | 11 | 10 | 8.3 | 16 | 11 | 9 | 14.3 | 13 | 12 |
| K56 | 12 | 10 | 11 | 14 | 11 | 9 | 12 | 15 | 11 | 11 | 13.3 | 14 | 12 |
| K57 | 11 | 9 | 15.3 | 14 | 12 | 10 | 12 | 15 | 11 | 9 | 13.3 | 15 | 10 |
| K58 | 11 | 12 | 17.3 | 14 | 11 | 12 | 13 | 14 | 11 | 11 | 13.3 | 12 | 14 |
| K59 | 11 | 10 | 14.3 | 15 | 10 | 10 | 8.3 | 17 | 11 | 11 | 15.3 | 14 | 14 |
| K60 | 11 | 10 | 15.3 | 17 | 11 | 8 | 12 | 16 | 11 | 9 | 14.3 | 15 | 12 |
| K61 | 9 | 10 | 15.3 | 14 | 11 | 10 | 8.3 | 16 | 9 | 11 | 13.3 | 14 | 12 |
| K62 | 10 | 13 | 17.3 | 15 | 11 | 10 | 8.3 | 15 | 11 | 12 | 13.3 | 12 | 14 |
| K63 | 12 | 11 | 12 | 16 | 10 | 11 | 12 | 16 | 11 | 9 | 14.3 | 13 | 14 |
| K64 | 10 | 12 | 12 | 15 | 10 | 6 | 8.3 | 15 | 10 | 11 | 13.3 | 14 | 13 |
| K65 | 12 | 11 | 13.3 | 15 | 10 | 11 | 12 | 16 | 10 | 9 | 13.3 | 15 | 12 |
| K66 | 12 | 10 | 15.3 | 15 | 11 | 8 | 8.3 | 15 | 11 | 13 | 12 | 14 | 14 |
| K67 | 12 | 11 | 14.3 | 14 | 11 | 11 | 11 | 14 | 11 | 11 | 13.3 | 14 | 13 |
| K68 | 11 | 10 | 15.3 | 16 | 12 | 12 | 8.3 | 16 | 10 | 11 | 13.3 | 14 | 10 |
| K69 | 10 | 10 | 16.3 | 14 | 11 | 8 | 8.3 | 17 | 12 | 10 | 13.3 | 14 | 13 |
| K70 | 11 | 10 | 14.3 | 15 | 10 | 12 | 12 | 18 | 9 | 13 | 13.3 | 14 | 14 |
| K71 | 11 | 12 | 15.3 | 14 | 10 | 11 | 13 | 14 | 10 | 11 | 15.3 | 14 | 16 |
| K72 | 11 | 10 | 17.3 | 13 | 12 | 10 | 11 | 15 | 7 | 11 | 16.3 | 13 | 13 |
| K73 | 12 | 11 | 14.3 | 15 | 10 | 11 | 8.3 | 15 | 12 | 13 | 14.3 | 15 | 14 |
| K74 | 11 | 10 | 14.3 | 15 | 11 | 8 | 12 | 15 | 9 | 11 | 14.3 | 13 | 15 |
| K75 | 12 | 9 | 12 | 15 | 11 | 10 | 12 | 10 | 7 | 10 | 15.3 | 14 | 14 |
| K76 | 11 | 11 | 14.3 | 14 | 10 | 6 | 8.3 | 18 | 9 | 9 | 15.3 | 13 | 14 |
| K77 | 10 | 11 | 15.3 | 15 | 10 | 11 | 8.3 | 15 | 10 | 11 | 12.3 | 12 | 13 |
| K78 | 11 | 12 | 15.3 | 14 | 11 | 11 | 11 | 13 | 9 | 13 | 13.3 | 15 | 13 |
| K79 | 12 | 11 | 16.3 | 14 | 11 | 9 | 13 | 16 | 10 | 11 | 13.3 | 14 | 15 |
| K80 | 10 | 11 | 15.3 | 14 | 10 | 10 | 8.3 | 15 | 10 | 11 | 14.3 | 15 | 12 |
| K81 | 11 | 10 | 14.3 | 15 | 10 | 11 | 12 | 14 | 10 | 13 | 12.3 | 13 | 12 |
| K82 | 10 | 11 | 14.3 | 17 | 11 | 9 | 8.3 | 16 | 12 | 11 | 14.3 | 12 | 13 |
| K83 | 11 | 11 | 13 | 15 | 10 | 12 | 13 | 16 | 8 | 13 | 14.3 | 14 | 14 |
| K84 | 11 | 8 | 15.3 | 17 | 11 | 11 | 14 | 15 | 10 | 9 | 13.3 | 13 | 13 |
| K85 | 10 | 10 | 15.3 | 17 | 11 | 10 | 12 | 17 | 9 | 9 | 13.3 | 13 | 12 |
| K86 | 10 | 11 | 15.3 | 13 | 10 | 11 | 12 | 16 | 11 | 9 | 13.3 | 14 | 13 |
| K87 | 8 | 10 | 15.3 | 15 | 10 | 10 | 8.3 | 16 | 10 | 11 | 13.3 | 12 | 12 |
| K88 | 10 | 11 | 13.3 | 15 | 10 | 10 | 8.3 | 17 | 9 | 9 | 13.3 | 13 | 14 |
| K89 | 10 | 12 | 17.3 | 17 | 10 | 10 | 13 | 13 | 7 | 9 | 14.3 | 13 | 13 |
| K90 | 12 | 9 | 15.3 | 15 | 11 | 11 | 8.3 | 15 | 7 | 9 | 14.3 | 14 | 13 |
| K91 | 11 | 10 | 15.3 | 16 | 12 | 10 | 8.3 | 15 | 11 | 11 | 12.3 | 14 | 13 |
| K92 | 11 | 11 | 16.3 | 14 | 10 | 11 | 12 | 16 | 11 | 10 | 16.3 | 15 | 14 |
| K93 | 12 | 11 | 15.3 | 14 | 11 | 7 | 8.3 | 16 | 11 | 11 | 15.3 | 12 | 14 |
| K94 | 10 | 8 | 17.3 | 14 | 11 | 8 | 12 | 16 | 11 | 13 | 13.3 | 12 | 12 |
| K95 | 10 | 10 | 14.3 | 15 | 11 | 10 | 13 | 15 | 9 | 13 | 13.3 | 14 | 13 |
| K96 | 10 | 11 | 12 | 16 | 11 | 10 | 8.3 | 17 | 9 | 13 | 16.3 | 15 | 13 |
| K97 | 11 | 11 | 17.3 | 14 | 11 | 10 | 8.3 | 15 | 11 | 11 | 14.3 | 14 | 13 |
| K98 | 10 | 11 | 15.3 | 13 | 9 | 10 | 8.3 | 11 | 7 | 11 | 13.3 | 15 | 12 |
| K99 | 12 | 11 | 16.3 | 14 | 12 | 12 | 11 | 15 | 12 | 11 | 15.3 | 13 | 14 |
| K100 | 11 | 11 | 11 | 14 | 10 | 10 | 8.3 | 16 | 9 | 11 | 14.3 | 12 | 14 |
| K101 | 11 | 11 | 16.3 | 15 | 11 | 10 | 12 | 15 | 7 | 11 | 13.3 | 12 | 13 |
| K102 | 11 | 10 | 12 | 16 | 10 | 9 | 12 | 11 | 7 | 9 | 13.3 | 12 | 14 |
| K103 | 10 | 11 | 15.3 | 14 | 11 | 12 | 8.3 | 14 | 9 | 13 | 13.3 | 15 | 13 |
| K104 | 10 | 10 | 15.3 | 14 | 11 | 6 | 12 | 15 | 11 | 13 | 15.3 | 14 | 13 |
| K105 | 11 | 10 | 15.3 | 15 | 12 | 11 | 12 | 11 | 10 | 9 | 13.3 | 16 | 12 |
| K106 | 10 | 11 | 15.3 | 15 | 11 | 9 | 8.3 | 16 | 7 | 9 | 14.3 | 15 | 14 |
| K107 | 10 | 12 | 17.3 | 17 | 10 | 6 | 8.3 | 12 | 7 | 9 | 13.3 | 15 | 13 |
| K108 | 11 | 11 | 10 | 15 | 10 | 10 | 12 | 15 | 13 | 11 | 13.3 | 15 | 14 |
| K109 | 11 | 12 | 16.3 | 14 | 11 | 8 | 8.3 | 17 | 7 | 13 | 13 | 14 | 13 |
| K110 | 11 | 10 | 15.3 | 14 | 10 | 12 | 8.3 | 16 | 11 | 10 | 15.3 | 14 | 13 |
| K111 | 11 | 10 | 15.3 | 16 | 11 | 10 | 12 | 16 | 10 | 11 | 14.3 | 13 | 13 |
| K112 | 12 | 10 | 17.3 | 14 | 9 | 9 | 8.3 | 15 | 11 | 13 | 12.3 | 12 | 14 |
| K113 | 10 | 11 | 15.3 | 14 | 12 | 6 | 8.3 | 15 | 10 | 9 | 12.3 | 13 | 14 |
| K114 | 11 | 11 | 15.3 | 14 | 11 | 10 | 8.3 | 16 | 11 | 11 | 16.3 | 15 | 13 |
| K115 | 12 | 10 | 16.3 | 14 | 11 | 6 | 12 | 15 | 9 | 11 | 15.3 | 13 | 13 |
| K116 | 12 | 10 | 15.3 | 14 | 10 | 10 | 8.3 | 14 | 7 | 9 | 12.3 | 16 | 11 |
| K117 | 11 | 10 | 14.3 | 15 | 10 | 8 | 12 | 15 | 9 | 9 | 13.3 | 14 | 12 |
| K118 | 11 | 11 | 15.3 | 14 | 11 | 11 | 12 | 16 | 11 | 10 | 12.3 | 15 | 14 |
| K119 | 11 | 11 | 15.3 | 15 | 11 | 11 | 14 | 13 | 11 | 11 | 12.3 | 14 | 11 |
| K120 | 12 | 11 | 15.3 | 15 | 12 | 12 | 8.3 | 16 | 7 | 9 | 12.3 | 15 | 14 |
| K121 | 11 | 11 | 12 | 15 | 11 | 11 | 8.3 | 13 | 7 | 13 | 13.3 | 14 | 12 |
| K122 | 11 | 12 | 12 | 15 | 13 | 10 | 12 | 16 | 10 | 11 | 12.3 | 14 | 14 |
| K123 | 11 | 10 | 12 | 15 | 10 | 10 | 8.3 | 15 | 7 | 9 | 12.3 | 13 | 14 |
| K124 | 11 | 11 | 14.3 | 17 | 10 | 8 | 12 | 15 | 12 | 11 | 14.3 | 14 | 14 |
| K125 | 12 | 11 | 15.3 | 14 | 11 | 11 | 8.3 | 15 | 10 | 9 | 16.3 | 15 | 13 |
| K126 | 11 | 10 | 14.3 | 14 | 11 | 12 | 12 | 15 | 9 | 13 | 13.3 | 12 | 13 |
| K127 | 12 | 11 | 14.3 | 16 | 10 | 11 | 14 | 13 | 10 | 9 | 12.3 | 14 | 12 |
| K128 | 10 | 11 | 15.3 | 15 | 10 | 12 | 12 | 12 | 10 | 9 | 15.3 | 12 | 12 |
| K129 | 10 | 10 | 14.3 | 14 | 11 | 6 | 13 | 14 | 12 | 11 | 15.3 | 12 | 12 |
| K130 | 9 | 10 | 16.3 | 14 | 10 | 11 | 11 | 16 | 12 | 11 | 13.3 | 14 | 13 |
| K131 | 11 | 8 | 15.3 | 16 | 10 | 10 | 8.3 | 16 | 7 | 11 | 15.3 | 15 | 16 |
| K132 | 11 | 11 | 15.3 | 15 | 10 | 12 | 13 | 15 | 7 | 11 | 14.3 | 14 | 15 |
| K133 | 11 | 10 | 15.3 | 14 | 12 | 10 | 8.3 | 17 | 7 | 11 | 13.3 | 14 | 14 |
| K134 | 10 | 11 | 14.3 | 15 | 10 | 12 | 13 | 16 | 8 | 11 | 13.3 | 15 | 13 |
| K135 | 12 | 10 | 14.3 | 14 | 10 | 11 | 12 | 16 | 10 | 10 | 14.3 | 14 | 13 |
| K136 | 11 | 10 | 13 | 15 | 10 | 11 | 8.3 | 16 | 10 | 13 | 13.3 | 13 | 12 |
| K137 | 11 | 11 | 15.3 | 14 | 11 | 10 | 13 | 15 | 11 | 9 | 13.3 | 13 | 12 |
| K138 | 12 | 11 | 15.3 | 16 | 12 | 6 | 8.3 | 13 | 11 | 11 | 16.3 | 14 | 13 |
| K139 | 11 | 10 | 15.3 | 15 | 10 | 10 | 8.3 | 11 | 9 | 11 | 14.3 | 15 | 13 |
| K140 | 10 | 11 | 13.3 | 15 | 11 | 10 | 8.3 | 17 | 9 | 11 | 12.3 | 14 | 15 |
| K141 | 11 | 11 | 14.3 | 15 | 10 | 10 | 12 | 17 | 10 | 11 | 13.3 | 14 | 15 |
| K142 | 10 | 11 | 11 | 17 | 12 | 11 | 8.3 | 16 | 10 | 9 | 12.3 | 13 | 13 |
| K143 | 11 | 10 | 15.3 | 14 | 11 | 6 | 14 | 15 | 10 | 11 | 13.3 | 14 | 15 |
| K144 | 12 | 10 | 15.3 | 16 | 12 | 11 | 13 | 16 | 11 | 10 | 13.3 | 13 | 13 |
| K145 | 10 | 10 | 14.3 | 14 | 11 | 8 | 12 | 13 | 7 | 13 | 14.3 | 15 | 12 |
| K146 | 10 | 11 | 14.3 | 15 | 10 | 8 | 11 | 15 | 9 | 9 | 15.3 | 15 | 13 |
| K147 | 10 | 10 | 16.3 | 15 | 11 | 12 | 12 | 15 | 13 | 13 | 14.3 | 14 | 14 |
| K148 | 12 | 10 | 14.3 | 14 | 11 | 8 | 8.3 | 13 | 10 | 13 | 13.3 | 14 | 14 |
| K149 | 10 | 10 | 15.3 | 14 | 10 | 6 | 11 | 17 | 11 | 13 | 13.3 | 13 | 13 |
| K150 | 11 | 10 | 14.3 | 15 | 11 | 6 | 14 | 14 | 10 | 11 | 13.3 | 16 | 14 |
| K151 | 10 | 10 | 15.3 | 17 | 10 | 8 | 12 | 15 | 9 | 12 | 14.3 | 15 | 13 |
| K152 | 10 | 11 | 15.3 | 14 | 12 | 10 | 13 | 13 | 9 | 11 | 15.3 | 15 | 14 |
| K153 | 11 | 12 | 15.3 | 14 | 12 | 11 | 8.3 | 14 | 10 | 13 | 13.3 | 13 | 14 |
| K154 | 11 | 11 | 16.3 | 14 | 10 | 12 | 11 | 12 | 10 | 11 | 13.3 | 14 | 13 |
| K155 | 12 | 10 | 15.3 | 14 | 11 | 10 | 8.3 | 17 | 9 | 11 | 15.3 | 15 | 12 |
| K156 | 12 | 11 | 12 | 14 | 12 | 12 | 13 | 16 | 12 | 11 | 13.3 | 14 | 11 |
| K157 | 11 | 11 | 15.3 | 15 | 12 | 11 | 8.3 | 15 | 10 | 11 | 13.3 | 13 | 12 |
| K158 | 12 | 10 | 11 | 15 | 10 | 6 | 13 | 13 | 10 | 13 | 14.3 | 13 | 15 |
| K159 | 12 | 13 | 16.3 | 15 | 11 | 11 | 8.3 | 15 | 11 | 9 | 13.3 | 13 | 13 |

**Supplementary Table S3:** Genetic profiles of the 13 X-STR loci were obtained from 66 unrelated Kurd females.

| **Sample name** | **DXS9902** | **DXS9902** | **DXS10164** | **DXS10164** | **DXS7130** | **DXS7130** | **DXS7423** | **DXS7423** | **DXS8378** | **DXS8378** | **GATA172D05** | **GATA172D05** |
| --- | --- | --- | --- | --- | --- | --- | --- | --- | --- | --- | --- | --- |
| fK1 | 11 | 11 | 10 | 10 | 15.3 | 15.3 | 14 | 15 | 11 | 12 | 8 | 11 |
| fK2 | 10 | 12 | 11 | 12 | 14 | 15.3 | 14 | 15 | 11 | 12 | 6 | 8 |
| fK3 | 10 | 12 | 10 | 10 | 14.3 | 15.3 | 14 | 15 | 10 | 11 | 8 | 9 |
| fK4 | 11 | 11 | 10 | 10 | 14.3 | 15.3 | 14 | 14 | 10 | 11 | 6 | 11 |
| fK5 | 10 | 10 | 10 | 12 | 12 | 16.3 | 13 | 16 | 6 | 12 | 10 | 12 |
| fK6 | 11 | 11 | 10 | 10 | 14.3 | 15.3 | 14 | 15 | 10 | 11 | 6 | 10 |
| fK7 | 10 | 11 | 10 | 11 | 11 | 14.3 | 15 | 15 | 10 | 12 | 10 | 11 |
| fK8 | 12 | 12 | 10 | 10 | 14.3 | 15.3 | 14 | 14 | 11 | 12 | 11 | 12 |
| fK9 | 12 | 12 | 10 | 11 | 14.3 | 15.3 | 14 | 15 | 10 | 11 | 8 | 11 |
| fK10 | 10 | 11 | 10 | 11 | 14.3 | 15.3 | 14 | 16 | 10 | 12 | 8 | 10 |
| fK11 | 10 | 12 | 10 | 12 | 14.3 | 15.3 | 14 | 14 | 11 | 12 | 6 | 12 |
| fK12 | 10 | 12 | 10 | 10 | 14.3 | 15.3 | 13 | 15 | 10 | 12 | 9 | 10 |
| fK13 | 10 | 10 | 10 | 11 | 14.3 | 15.3 | 14 | 15 | 11 | 11 | 8 | 10 |
| fK14 | 11 | 11 | 11 | 11 | 14.3 | 15.3 | 15 | 15 | 10 | 11 | 10 | 10 |
| fK15 | 12 | 12 | 10 | 10 | 15.3 | 15.3 | 15 | 16 | 11 | 12 | 10 | 10 |
| fK16 | 10 | 10 | 10 | 11 | 14.3 | 15.3 | 16 | 17 | 10 | 10 | 11 | 11 |
| fK17 | 10 | 10 | 10 | 11 | 15.3 | 15.3 | 14 | 15 | 10 | 11 | 8 | 10 |
| fK18 | 10 | 11 | 10 | 10 | 15.3 | 15.3 | 14 | 15 | 11 | 11 | 11 | 11 |
| fK19 | 10 | 11 | 10 | 11 | 14.3 | 15.3 | 15 | 16 | 11 | 12 | 8 | 11 |
| fK20 | 11 | 12 | 10 | 10 | 15.3 | 15.3 | 16 | 17 | 10 | 10 | 10 | 10 |
| fK21 | 9 | 12 | 10 | 10 | 15.3 | 16.3 | 15 | 16 | 11 | 12 | 10 | 11 |
| fK22 | 11 | 11 | 10 | 13 | 14.3 | 15.3 | 15 | 15 | 10 | 10 | 6 | 9 |
| fK23 | 10 | 11 | 10 | 10 | 11 | 14.3 | 14 | 15 | 10 | 11 | 6 | 10 |
| fK24 | 10 | 11 | 10 | 10 | 14.3 | 16.3 | 14 | 15 | 10 | 11 | 6 | 11 |
| fK25 | 10 | 11 | 10 | 10 | 15.3 | 15.3 | 14 | 15 | 10 | 10 | 6 | 11 |
| fK26 | 11 | 12 | 10 | 10 | 14.3 | 15.3 | 14 | 15 | 10 | 12 | 10 | 11 |
| fK27 | 11 | 11 | 9 | 10 | 14.3 | 15.3 | 14 | 16 | 10 | 12 | 8 | 10 |
| fK28 | 10 | 11 | 10 | 10 | 14.3 | 15.3 | 14 | 16 | 10 | 11 | 12 | 12 |
| fK29 | 10 | 12 | 12 | 13 | 14.3 | 14.3 | 14 | 15 | 11 | 11 | 8 | 10 |
| fK30 | 10 | 11 | 10 | 10 | 15.3 | 15.3 | 15 | 15 | 11 | 13 | 6 | 12 |
| fK31 | 9 | 11 | 10 | 10 | 12 | 17.3 | 14 | 15 | 10 | 11 | 8 | 11 |
| fK32 | 11 | 12 | 10 | 10 | 12 | 16.3 | 14 | 16 | 10 | 12 | 8 | 10 |
| fK33 | 10 | 10 | 11 | 12 | 15.3 | 15.3 | 14 | 16 | 10 | 11 | 6 | 12 |
| fK34 | 10 | 11 | 10 | 10 | 15.3 | 15.3 | 14 | 15 | 11 | 11 | 11 | 11 |
| fK35 | 10 | 11 | 11 | 12 | 14.3 | 14.3 | 14 | 16 | 11 | 12 | 6 | 12 |
| fK36 | 10 | 12 | 9 | 10 | 15.3 | 16.3 | 14 | 17 | 10 | 10 | 8 | 10 |
| fK37 | 11 | 12 | 10 | 10 | 14.3 | 15.3 | 15 | 16 | 10 | 11 | 8 | 12 |
| fK38 | 11 | 11 | 10 | 11 | 14.3 | 14.3 | 14 | 15 | 8 | 8 | 9 | 12 |
| fK39 | 10 | 11 | 10 | 11 | 15.3 | 16.3 | 13 | 14 | 10 | 11 | 8 | 12 |
| fK40 | 12 | 12 | 10 | 10 | 13 | 14.3 | 15 | 17 | 12 | 13 | 9 | 11 |
| fK41 | 10 | 11 | 8 | 11 | 15.3 | 15.3 | 15 | 15 | 12 | 12 | 11 | 12 |
| fK42 | 11 | 12 | 11 | 12 | 13 | 14.3 | 14 | 15 | 11 | 11 | 6 | 8 |
| fK43 | 10 | 10 | 9 | 12 | 13 | 15.3 | 14 | 15 | 10 | 10 | 10 | 10 |
| fK44 | 11 | 12 | 10 | 10 | 12 | 15.3 | 13 | 15 | 11 | 11 | 10 | 11 |
| fK45 | 11 | 12 | 10 | 11 | 15.3 | 15.3 | 14 | 15 | 11 | 12 | 8 | 9 |
| fK46 | 11 | 11 | 10 | 10 | 14.3 | 15.3 | 14 | 17 | 10 | 12 | 8 | 12 |
| fK47 | 10 | 11 | 12 | 12 | 14.3 | 16.3 | 14 | 15 | 11 | 11 | 8 | 13 |
| fK48 | 10 | 11 | 10 | 11 | 14.3 | 14.3 | 15 | 16 | 10 | 10 | 10 | 12 |
| fK49 | 11 | 11 | 11 | 12 | 13 | 14.3 | 14 | 16 | 10 | 11 | 8 | 10 |
| fK50 | 12 | 12 | 10 | 11 | 12 | 15.3 | 14 | 15 | 11 | 12 | 6 | 11 |
| fK51 | 11 | 11 | 10 | 12 | 14.3 | 15.3 | 14 | 15 | 10 | 12 | 8 | 10 |
| fK52 | 11 | 11 | 10 | 11 | 14.3 | 15.3 | 14 | 16 | 10 | 11 | 11 | 12 |
| fK53 | 10 | 12 | 10 | 11 | 15.3 | 16.3 | 15 | 15 | 10 | 11 | 6 | 10 |
| fK54 | 11 | 11 | 10 | 11 | 15.3 | 16.3 | 15 | 16 | 11 | 11 | 6 | 12 |
| fK55 | 10 | 11 | 11 | 11 | 13 | 15.3 | 15 | 15 | 10 | 10 | 6 | 12 |
| fK56 | 11 | 12 | 10 | 10 | 14.3 | 14.3 | 14 | 15 | 11 | 12 | 6 | 10 |
| fK57 | 11 | 12 | 11 | 12 | 17 | 18 | 14 | 15 | 10 | 11 | 6 | 9 |
| fK58 | 10 | 11 | 9 | 10 | 15.3 | 15.3 | 14 | 15 | 10 | 11 | 10 | 10 |
| fK59 | 10 | 10 | 10 | 12 | 12 | 16.3 | 13 | 16 | 10 | 10 | 10 | 12 |
| fK60 | 11 | 11 | 10 | 11 | 14.3 | 15.3 | 14 | 17 | 10 | 10 | 6 | 12 |
| fK61 | 10 | 11 | 10 | 11 | 14.3 | 15.3 | 14 | 15 | 11 | 12 | 8 | 10 |
| fK62 | 12 | 12 | 10 | 12 | 12 | 14.3 | 14 | 15 | 10 | 12 | 11 | 12 |
| fK63 | 10 | 11 | 10 | 11 | 14.3 | 15.3 | 14 | 15 | 10 | 13 | 6 | 11 |
| fK64 | 10 | 11 | 10 | 10 | 14.3 | 15.3 | 14 | 14 | 10 | 12 | 6 | 12 |
| fK65 | 10 | 10 | 10 | 11 | 16.3 | 16.3 | 14 | 16 | 10 | 10 | 12 | 12 |
| fK66 | 11 | 12 | 11 | 14 | 14.3 | 15.3 | 14 | 14 | 11 | 11 | 6 | 10 |

| **Sample name** | **DXS9898** | **DXS9898** | **DXS7424** | **DXS7424** | **GATA31E08** | **GATA31E08** | **DXS6795** | **DXS6795** | **DXS981** | **DXS981** | **DXS7132** | **DXS7132** | **GATA144D04** | **GATA144D04** |
| --- | --- | --- | --- | --- | --- | --- | --- | --- | --- | --- | --- | --- | --- | --- |
| fK1 | 8.3 | 12 | 15 | 16 | 10 | 11 | 9 | 13 | 13.3 | 13.3 | 13 | 14 | 11 | 14 |
| fK2 | 8.3 | 12 | 15 | 17 | 9 | 9 | 13 | 13 | 13.3 | 13.3 | 12 | 14 | 12 | 13 |
| fK3 | 8.3 | 13 | 15 | 15 | 9 | 10 | 9 | 13 | 13.3 | 14.3 | 13 | 15 | 13 | 14 |
| fK4 | 8.3 | 11 | 14 | 14 | 7 | 10 | 9 | 13 | 13.3 | 14.3 | 13 | 14 | 13 | 14 |
| fK5 | 13 | 13 | 14 | 17 | 10 | 11 | 9 | 11 | 15.3 | 16.3 | 14 | 14 | 12 | 15 |
| fK6 | 8.3 | 8.3 | 10 | 17 | 10 | 11 | 11 | 11 | 13.3 | 14.3 | 12 | 15 | 11 | 12 |
| fK7 | 8.3 | 13 | 15 | 17 | 7 | 9 | 11 | 11 | 13.3 | 13.3 | 13 | 14 | 11 | 13 |
| fK8 | 8.3 | 11 | 15 | 16 | 11 | 11 | 9 | 9 | 13.3 | 13.3 | 14 | 14 | 11 | 14 |
| fK9 | 8.3 | 11 | 15 | 15 | 7 | 10 | 9 | 9 | 13.3 | 13.3 | 13 | 15 | 12 | 12 |
| fK10 | 12 | 12 | 15 | 16 | 7 | 9 | 9 | 11 | 14.3 | 14.3 | 14 | 14 | 12 | 13 |
| fK11 | 11 | 11 | 15 | 16 | 10 | 10 | 10 | 12 | 13.3 | 14.3 | 13 | 14 | 11 | 14 |
| fK12 | 8.3 | 12 | 15 | 16 | 7 | 9 | 11 | 11 | 14.3 | 14.3 | 14 | 14 | 14 | 14 |
| fK13 | 8.3 | 8.3 | 15 | 16 | 8 | 11 | 11 | 11 | 13.3 | 14.3 | 14 | 16 | 13 | 14 |
| fK14 | 8.3 | 8.3 | 12 | 16 | 11 | 11 | 11 | 12 | 13.3 | 14.3 | 14 | 15 | 13 | 14 |
| fK15 | 8.3 | 11 | 14 | 15 | 11 | 13 | 10 | 12 | 13.3 | 14.3 | 12 | 14 | 13 | 13 |
| fK16 | 11 | 13 | 14 | 15 | 9 | 10 | 10 | 13 | 13.3 | 14.3 | 13 | 13 | 12 | 13 |
| fK17 | 8.3 | 11 | 14 | 16 | 12 | 12 | 11 | 11 | 13.3 | 13.3 | 13 | 14 | 13 | 13 |
| fK18 | 11 | 11 | 15 | 16 | 7 | 10 | 11 | 12 | 14.3 | 14.3 | 13 | 14 | 13 | 15 |
| fK19 | 8.3 | 12 | 16 | 17 | 9 | 10 | 9 | 10 | 12.3 | 13.3 | 12 | 13 | 12 | 15 |
| fK20 | 8.3 | 13 | 14 | 15 | 9 | 11 | 9 | 13 | 14.3 | 14.3 | 13 | 14 | 12 | 13 |
| fK21 | 12 | 13 | 14 | 15 | 9 | 9 | 11 | 13 | 12.3 | 15.3 | 13 | 14 | 12 | 13 |
| fK22 | 11 | 11 | 13 | 16 | 10 | 10 | 9 | 11 | 14.3 | 14.3 | 15 | 15 | 12 | 13 |
| fK23 | 11 | 12 | 16 | 17 | 10 | 12 | 11 | 11 | 14.3 | 16.3 | 14 | 15 | 13 | 13 |
| fK24 | 13 | 13 | 15 | 16 | 10 | 12 | 11 | 11 | 12.3 | 13.3 | 13 | 15 | 12 | 16 |
| fK25 | 11 | 12 | 12 | 17 | 7 | 9 | 12 | 13 | 13.3 | 14.3 | 12 | 14 | 10 | 13 |
| fK26 | 8.3 | 12 | 16 | 16 | 10 | 10 | 11 | 12 | 13.3 | 14.3 | 14 | 15 | 12 | 14 |
| fK27 | 8.3 | 14 | 16 | 16 | 7 | 7 | 9 | 13 | 13.3 | 14.3 | 13 | 14 | 12 | 12 |
| fK28 | 11 | 13 | 15 | 16 | 7 | 9 | 11 | 11 | 13.3 | 13.3 | 13 | 14 | 12 | 13 |
| fK29 | 8.3 | 12 | 14 | 17 | 9 | 11 | 9 | 11 | 13.3 | 14.3 | 14 | 14 | 13 | 13 |
| fK30 | 8.3 | 11 | 16 | 17 | 9 | 11 | 11 | 11 | 14.3 | 15.3 | 12 | 14 | 14 | 14 |
| fK31 | 11 | 11 | 15 | 16 | 7 | 10 | 11 | 11 | 13.3 | 15.3 | 13 | 13 | 10 | 12 |
| fK32 | 12 | 12 | 14 | 15 | 7 | 10 | 9 | 13 | 14.3 | 15.3 | 13 | 14 | 14 | 14 |
| fK33 | 11 | 12 | 15 | 16 | 9 | 10 | 11 | 13 | 13.3 | 13.3 | 13 | 14 | 13 | 14 |
| fK34 | 11 | 11 | 15 | 16 | 7 | 10 | 9 | 11 | 14.3 | 14.3 | 13 | 14 | 13 | 15 |
| fK35 | 8.3 | 12 | 14 | 16 | 10 | 10 | 10 | 11 | 13.3 | 14.3 | 14 | 15 | 13 | 15 |
| fK36 | 8.3 | 14 | 15 | 16 | 7 | 10 | 9 | 11 | 13.3 | 13.3 | 14 | 14 | 13 | 16 |
| fK37 | 11 | 12 | 14 | 15 | 9 | 9 | 9 | 11 | 13.3 | 15.3 | 13 | 14 | 14 | 14 |
| fK38 | 11 | 13 | 13 | 15 | 7 | 10 | 11 | 11 | 14.3 | 14.3 | 14 | 14 | 13 | 16 |
| fK39 | 8.3 | 14 | 14 | 16 | 9 | 11 | 9 | 11 | 13.3 | 13.3 | 15 | 15 | 13 | 13 |
| fK40 | 11 | 11 | 14 | 15 | 7 | 10 | 9 | 13 | 13.3 | 13.3 | 14 | 15 | 13 | 15 |
| fK41 | 8.3 | 12 | 16 | 17 | 10 | 11 | 11 | 13 | 13.3 | 13.3 | 13 | 13 | 14 | 15 |
| fK42 | 8.3 | 8.3 | 13 | 15 | 7 | 11 | 9 | 13 | 13.3 | 15.3 | 14 | 14 | 12 | 12 |
| fK43 | 8.3 | 12 | 13 | 17 | 11 | 11 | 9 | 11 | 13.3 | 13.3 | 13 | 14 | 10 | 13 |
| fK44 | 11 | 11 | 16 | 16 | 11 | 12 | 9 | 11 | 13.3 | 14.3 | 12 | 14 | 13 | 15 |
| fK45 | 8.3 | 13 | 13 | 16 | 10 | 11 | 9 | 11 | 13.3 | 14.3 | 13 | 14 | 13 | 14 |
| fK46 | 8.3 | 11 | 13 | 16 | 9 | 11 | 10 | 13 | 13.3 | 15.3 | 13 | 13 | 13 | 14 |
| fK47 | 13 | 13 | 15 | 16 | 11 | 11 | 9 | 11 | 14.3 | 16.3 | 12 | 13 | 13 | 15 |
| fK48 | 8.3 | 13 | 15 | 16 | 9 | 11 | 9 | 11 | 13.3 | 14.3 | 13 | 14 | 12 | 13 |
| fK49 | 8.3 | 8.3 | 16 | 17 | 9 | 10 | 11 | 13 | 13.3 | 13.3 | 13 | 15 | 14 | 16 |
| fK50 | 12 | 12 | 15 | 15 | 7 | 10 | 9 | 9 | 14.3 | 14.3 | 13 | 15 | 13 | 15 |
| fK51 | 8.3 | 8.3 | 15 | 16 | 12 | 12 | 11 | 13 | 13.3 | 15.3 | 13 | 14 | 13 | 13 |
| fK52 | 8.3 | 8.3 | 15 | 16 | 7 | 9 | 10 | 11 | 14.3 | 14.3 | 13 | 15 | 12 | 13 |
| fK53 | 8.3 | 12 | 15 | 16 | 7 | 9 | 10 | 10 | 12.3 | 15.3 | 13 | 15 | 13 | 14 |
| fK54 | 8.3 | 11 | 13 | 15 | 7 | 7 | 11 | 13 | 13.3 | 13.3 | 13 | 13 | 15 | 15 |
| fK55 | 8.3 | 12 | 10 | 12 | 11 | 12 | 11 | 11 | 13.3 | 16.3 | 13 | 15 | 10 | 13 |
| fK56 | 8.3 | 12 | 13 | 15 | 10 | 11 | 9 | 11 | 13.3 | 14.3 | 13 | 13 | 12 | 13 |
| fK57 | 12 | 13 | 15 | 16 | 7 | 11 | 11 | 12 | 13.3 | 13.3 | 12 | 14 | 13 | 13 |
| fK58 | 8.3 | 12 | 14 | 17 | 10 | 11 | 9 | 11 | 13.3 | 13.3 | 15 | 15 | 13 | 13 |
| fK59 | 13 | 13 | 14 | 17 | 10 | 11 | 9 | 11 | 15.3 | 16.3 | 14 | 14 | 12 | 15 |
| fK60 | 8.3 | 12 | 15 | 18 | 7 | 10 | 9 | 11 | 13.3 | 14.3 | 14 | 15 | 12 | 12 |
| fK61 | 8.3 | 13 | 14 | 15 | 10 | 12 | 11 | 11 | 13.3 | 15.3 | 14 | 15 | 11 | 13 |
| fK62 | 11 | 14 | 12 | 15 | 7 | 11 | 11 | 11 | 13.3 | 13.3 | 13 | 15 | 13 | 14 |
| fK63 | 8.3 | 12 | 13 | 15 | 7 | 9 | 9 | 9 | 13.3 | 14.3 | 14 | 15 | 13 | 14 |
| fK64 | 8.3 | 12 | 14 | 15 | 9 | 10 | 11 | 11 | 13.3 | 16.3 | 13 | 15 | 12 | 13 |
| fK65 | 11 | 11 | 13 | 16 | 9 | 11 | 11 | 11 | 14.3 | 16.3 | 15 | 15 | 15 | 16 |
| fK66 | 8.3 | 8.3 | 15 | 16 | 7 | 12 | 11 | 13 | 13.3 | 14.3 | 14 | 14 | 13 | 13 |

**Supplementary Table S4:** Allele frequencies of the 13 X-STR loci using female (F), male (M), and pool (P) data.

| **Allele** | **DXS9902-F** | **DXS9902-M** | **DXS9902-P** | **DXS10164-F** | **DXS10164-M** | **DXS10164-P** | **DXS7130-F** | **DXS7130-M** | **DXS7130-P** | **DXS7423-F** | **DXS7423-M** | **DXS7423-P** |
| --- | --- | --- | --- | --- | --- | --- | --- | --- | --- | --- | --- | --- |
| **5** |  |  |  |  |  |  |  |  |  |  |  |  |
| **6** |  |  |  |  |  |  |  |  |  |  |  |  |
| **7** |  |  |  |  |  |  |  |  |  |  |  |  |
| **8** |  | 0.0063 | 0.0034 | 0.0076 | 0.0252 | 0.0172 |  |  |  |  |  |  |
| **8.3** |  |  |  |  |  |  |  |  |  |  |  |  |
| **9** | 0.0152 | 0.0252 | 0.0206 | 0.0303 | 0.0252 | 0.0275 |  |  |  |  |  |  |
| **10** | 0.3258 | 0.327 | 0.3265 | 0.5909 | 0.4214 | 0.4983 |  | 0.0063 | 0.0034 |  |  |  |
| **11** | 0.4318 | 0.3899 | 0.4089 | 0.2348 | 0.4025 | 0.3265 | 0.0152 | 0.0314 | 0.0241 |  |  |  |
| **12** | 0.2273 | 0.2516 | 0.2405 | 0.1136 | 0.1069 | 0.11 | 0.053 | 0.0692 | 0.0619 |  |  |  |
| **12.3** |  |  |  |  |  |  |  |  |  |  |  |  |
| **13** |  |  |  | 0.0152 | 0.0126 | 0.0137 | 0.0379 | 0.0189 | 0.0275 | 0.0379 | 0.0252 | 0.0309 |
| **13.3** |  |  |  |  |  |  |  | 0.0252 | 0.0137 |  |  |  |
| **14** |  |  |  | 0.0076 |  | 0.0034 | 0.0076 |  | 0.0034 | 0.3864 | 0.4025 | 0.3952 |
| **14.3** |  |  |  |  |  |  | 0.3258 | 0.2264 | 0.2715 |  |  |  |
| **15** |  |  |  |  | 0.0063 | 0.0034 |  |  |  | 0.3864 | 0.3648 | 0.3746 |
| **15.3** |  |  |  |  |  |  | 0.447 | 0.4717 | 0.4605 |  |  |  |
| **16** |  |  |  |  |  |  |  |  |  | 0.1439 | 0.1069 | 0.1237 |
| **16.3** |  |  |  |  |  |  | 0.0909 | 0.0943 | 0.0928 |  |  |  |
| **17** |  |  |  |  |  |  | 0.0076 |  | 0.0034 | 0.0455 | 0.1006 | 0.0756 |
| **17.3** |  |  |  |  |  |  | 0.0076 | 0.0566 | 0.0344 |  |  |  |
| **18** |  |  |  |  |  |  | 0.0076 |  | 0.0034 |  |  |  |

| **Allele** | **DXS8378-F** | **DXS8378-M** | **DXS8378-P** | **GATA172D05-F** | **GATA172D05-M** | **GATA172D05-P** | **DXS9898-F** | **DXS9898-M** | **DXS9898-P** | **DXS7424-F** | **DXS7424-M** | **DXS7424-P** |
| --- | --- | --- | --- | --- | --- | --- | --- | --- | --- | --- | --- | --- |
| **5** |  |  |  |  | 0.0063 | 0.0034 |  |  |  |  |  |  |
| **6** | 0.0076 | 0.0126 | 0.0103 | 0.1667 | 0.1635 | 0.1649 |  |  |  |  |  |  |
| **7** |  |  |  |  | 0.0063 | 0.0034 |  |  |  |  |  |  |
| **8** | 0.0152 |  | 0.0069 | 0.1667 | 0.1384 | 0.1512 |  |  |  |  |  |  |
| **8.3** |  |  |  |  |  |  | 0.3712 | 0.3962 | 0.3849 |  |  |  |
| **9** |  | 0.0189 | 0.0103 | 0.053 | 0.0818 | 0.0687 |  |  |  |  |  |  |
| **10** | 0.3864 | 0.3396 | 0.3608 | 0.25 | 0.283 | 0.268 |  | 0.0063 | 0.0034 | 0.0152 | 0.0252 | 0.0206 |
| **11** | 0.3712 | 0.4088 | 0.3918 | 0.1818 | 0.195 | 0.189 | 0.2424 | 0.1321 | 0.1821 |  | 0.0314 | 0.0172 |
| **12** | 0.197 | 0.2138 | 0.2062 | 0.1742 | 0.1258 | 0.1478 | 0.2121 | 0.3019 | 0.2612 | 0.0303 | 0.0377 | 0.0344 |
| **12.3** |  |  |  |  |  |  |  |  |  |  |  |  |
| **13** | 0.0227 | 0.0063 | 0.0137 | 0.0076 |  | 0.0034 | 0.1439 | 0.1321 | 0.1375 | 0.0758 | 0.1006 | 0.0893 |
| **13.3** |  |  |  |  |  |  |  |  |  |  |  |  |
| **14** |  |  |  |  |  |  | 0.0303 | 0.0314 | 0.0309 | 0.1364 | 0.1132 | 0.1237 |
| **14.3** |  |  |  |  |  |  |  |  |  |  |  |  |
| **15** |  |  |  |  |  |  |  |  |  | 0.3333 | 0.3208 | 0.3265 |
| **15.3** |  |  |  |  |  |  |  |  |  |  |  |  |
| **16** |  |  |  |  |  |  |  |  |  | 0.2955 | 0.2767 | 0.2852 |
| **16.3** |  |  |  |  |  |  |  |  |  |  |  |  |
| **17** |  |  |  |  |  |  |  |  |  | 0.1061 | 0.0818 | 0.0928 |
| **17.3** |  |  |  |  |  |  |  |  |  |  |  |  |
| **18** |  |  |  |  |  |  |  |  |  | 0.0076 | 0.0126 | 0.0103 |

| **Allele** | **GATA31E08-F** | **GATA31E08-M** | **GATA31E08-P** | **DXS6795-F** | **DXS6795-M** | **DXS6795-P** | **DXS981-F** | **DXS981-M** | **DXS981-P** | **DXS7132-F** | **DXS7132-M** | **DXS7132-P** | **GATA144D04-F** | **GATA144D04-M** | **GATA144D04-P** |
| --- | --- | --- | --- | --- | --- | --- | --- | --- | --- | --- | --- | --- | --- | --- | --- |
| **5** |  |  |  |  |  |  |  |  |  |  |  |  |  |  |  |
| **6** |  |  |  |  |  |  |  |  |  |  |  |  |  |  |  |
| **7** | 0.2045 | 0.1824 | 0.1924 |  |  |  |  |  |  |  |  |  |  |  |  |
| **8** | 0.0076 | 0.0126 | 0.0103 |  |  |  |  |  |  |  |  |  |  |  |  |
| **8.3** |  |  |  |  |  |  |  |  |  |  |  |  |  |  |  |
| **9** | 0.2045 | 0.2201 | 0.2131 | 0.2576 | 0.283 | 0.2715 |  |  |  |  |  |  |  |  |  |
| **10** | 0.2727 | 0.2453 | 0.2577 | 0.0682 | 0.0629 | 0.0653 |  |  |  |  |  |  | 0.0303 | 0.0252 | 0.0275 |
| **11** | 0.2273 | 0.2516 | 0.2405 | 0.4697 | 0.434 | 0.4502 |  |  |  |  |  |  | 0.0455 | 0.0252 | 0.0344 |
| **12** | 0.0758 | 0.0755 | 0.0756 | 0.053 | 0.0126 | 0.0309 |  | 0.0063 | 0.0034 | 0.0682 | 0.1258 | 0.0997 | 0.197 | 0.1824 | 0.189 |
| **12.3** |  |  |  |  |  |  | 0.0303 | 0.1447 | 0.0928 |  |  |  |  |  |  |
| **13** | 0.0076 | 0.0126 | 0.0103 | 0.1515 | 0.2013 | 0.1787 |  | 0.0063 | 0.0034 | 0.3182 | 0.2516 | 0.2818 | 0.4015 | 0.4025 | 0.4021 |
| **13.3** |  |  |  |  |  |  | 0.5 | 0.4654 | 0.4811 |  |  |  |  |  |  |
| **14** |  |  |  |  | 0.0063 | 0.0034 |  |  |  | 0.4015 | 0.3774 | 0.3883 | 0.1818 | 0.2579 | 0.2234 |
| **14.3** |  |  |  |  |  |  | 0.3258 | 0.2138 | 0.2646 |  |  |  |  |  |  |
| **15** |  |  |  |  |  |  |  |  |  | 0.2045 | 0.2201 | 0.2131 | 0.1061 | 0.0881 | 0.0962 |
| **15.3** |  |  |  |  |  |  | 0.0909 | 0.1195 | 0.1065 |  |  |  |  |  |  |
| **16** |  |  |  |  |  |  |  |  |  | 0.0076 | 0.0252 | 0.0172 | 0.0379 | 0.0189 | 0.0275 |
| **16.3** |  |  |  |  |  |  | 0.053 | 0.044 | 0.0481 |  |  |  |  |  |  |
| **17** |  |  |  |  |  |  |  |  |  |  |  |  |  |  |  |
| **17.3** |  |  |  |  |  |  |  |  |  |  |  |  |  |  |  |
| **18** |  |  |  |  |  |  |  |  |  |  |  |  |  |  |  |

**Supplementary Table S5:** Forensic parameters, Gene diversity (GD), polymorphism information content (PIC), match probability (PM), power of discrimination (PD), observed heterozygosity (Hobs), and power of exclusion (PE) were calculated for the in-house 13 X-STR loci in the Kurdish female using the STRAF online tool.

| locus | N | GD | PIC | PM | PD | Hobs | PE |
| --- | --- | --- | --- | --- | --- | --- | --- |
| DXS10164 | 132 | 0.58594 | 0.53148 | 0.24472 | 0.75528 | 0.57576 | 0.26279 |
| DXS6795 | 132 | 0.68783 | 0.63552 | 0.16208 | 0.83792 | 0.66667 | 0.3786 |
| DXS7130 | 132 | 0.68633 | 0.6307 | 0.19697 | 0.80303 | 0.72727 | 0.4717 |
| DXS7132 | 132 | 0.69628 | 0.63353 | 0.15657 | 0.84343 | 0.69697 | 0.42359 |
| DXS7423 | 132 | 0.6824 | 0.61805 | 0.22911 | 0.77089 | 0.81818 | 0.63321 |
| DXS7424 | 132 | 0.77065 | 0.72996 | 0.1382 | 0.8618 | 0.89394 | 0.78306 |
| DXS8378 | 132 | 0.67846 | 0.60938 | 0.17815 | 0.82185 | 0.66667 | 0.3786 |
| DXS981 | 132 | 0.63671 | 0.57022 | 0.19972 | 0.80028 | 0.59091 | 0.28011 |
| DXS9898 | 132 | 0.74242 | 0.69242 | 0.1258 | 0.8742 | 0.65152 | 0.3573 |
| DXS9902 | 132 | 0.66054 | 0.58557 | 0.18825 | 0.81175 | 0.59091 | 0.28011 |
| GATA144D04 | 132 | 0.757 | 0.71811 | 0.0978 | 0.9022 | 0.72727 | 0.4717 |
| GATA172D05 | 132 | 0.82188 | 0.78915 | 0.07668 | 0.92332 | 0.84848 | 0.69188 |
| GATA31E08 | 132 | 0.79042 | 0.7497 | 0.09366 | 0.90634 | 0.77273 | 0.54944 |

**Supplementary Table S6:** Forensic parameters, Gene diversity (GD), polymorphism information content (PIC), match probability (PM), and power of discrimination (PD) were calculated for the in-house 13 X-STR loci in the Kurdish male using the STRAF online tool.

| locus | N | GD | PIC | PM | PD |
| --- | --- | --- | --- | --- | --- |
| DXS10164 | 159 | 0.65162 | 0.58119 | 0.35248 | 0.64752 |
| DXS6795 | 159 | 0.69127 | 0.63244 | 0.31308 | 0.68692 |
| DXS7130 | 159 | 0.71181 | 0.67392 | 0.29267 | 0.70733 |
| DXS7132 | 159 | 0.73402 | 0.68306 | 0.2706 | 0.7294 |
| DXS7423 | 159 | 0.68705 | 0.62625 | 0.31727 | 0.68273 |
| DXS7424 | 159 | 0.79269 | 0.7594 | 0.21229 | 0.78771 |
| DXS8378 | 159 | 0.6755 | 0.60651 | 0.32874 | 0.67126 |
| DXS981 | 159 | 0.70488 | 0.66037 | 0.29955 | 0.70045 |
| DXS9898 | 159 | 0.72048 | 0.66883 | 0.28405 | 0.71595 |
| DXS9902 | 159 | 0.68132 | 0.61129 | 0.32297 | 0.67703 |
| GATA144D04 | 159 | 0.73346 | 0.68716 | 0.27115 | 0.72885 |
| GATA172D05 | 159 | 0.81857 | 0.78784 | 0.18658 | 0.81342 |
| GATA31E08 | 159 | 0.79381 | 0.75533 | 0.21119 | 0.78881 |

**Supplementary Table S7:** Number of alleles, power of discrimination, Combined power of discrimination, mean exclusion chance (MEC_kruger, MEC_Kishida, MEC_Desmarais, and MEC_Desmarais Duo) and combined MEC of the 13 X-STR loci using StatsX v2.0.

| Forensic Parameters | DXS9902 | DXS10164 | DXS7130 | DXS7423 | DXS8378 | GATA172D05 | DXS9898 |
| --- | --- | --- | --- | --- | --- | --- | --- |
| N_Haplotypes/Alleles | 5 | 8 | 12 | 5 | 7 | 9 | 6 |
| HD_GD | 0.67023 | 0.63391 | 0.70144 | 0.68387 | 0.67567 | 0.81862 | 0.73311 |
| PIC | 0.60031 | 0.56928 | 0.65894 | 0.62444 | 0.60897 | 0.79007 | 0.68607 |
| PD_Male | 0.66792 | 0.63173 | 0.69903 | 0.68152 | 0.67335 | 0.8158 | 0.73059 |
| PD_Female | 0.82211 | 0.80192 | 0.86933 | 0.84149 | 0.82892 | 0.94034 | 0.8829 |
| MEC_Kruger | 0.38642 | 0.37175 | 0.47734 | 0.42583 | 0.40022 | 0.63244 | 0.49341 |
| MEC_Kishida | 0.60019 | 0.56928 | 0.65894 | 0.62444 | 0.60897 | 0.78984 | 0.68607 |
| MEC_Desmarais | 0.60031 | 0.56928 | 0.65894 | 0.62444 | 0.60897 | 0.79007 | 0.68607 |
| MEC_Desmarais_duo | 0.45294 | 0.42336 | 0.51684 | 0.47968 | 0.46258 | 0.6691 | 0.5447 |

| Forensic Parameters | DXS9898 | DXS7424 | GATA31E08 | DXS6795 | DXS981 | DXS7132 | GATA144D04 | Combined |
| --- | --- | --- | --- | --- | --- | --- | --- | --- |
| N_Haplotypes/Alleles | 6 | 9 | 7 | 6 | 7 | 5 | 7 | - |
| HD_GD | 0.73311 | 0.78084 | 0.79011 | 0.68881 | 0.67857 | 0.71663 | 0.74329 | - |
| PIC | 0.68607 | 0.7473 | 0.75341 | 0.63568 | 0.6301 | 0.66366 | 0.70352 | - |
| PD_Male | 0.73059 | 0.77816 | 0.78739 | 0.68644 | 0.67624 | 0.71416 | 0.74074 | 0.99999993257 |
| PD_Female | 0.8829 | 0.91993 | 0.92082 | 0.85092 | 0.84904 | 0.8678 | 0.89556 | 0.9999999999982 |
| MEC_Kruger | 0.49341 | 0.58006 | 0.57781 | 0.43849 | 0.43902 | 0.46442 | 0.52232 | 0.9998154134240 |
| MEC_Kishida | 0.68607 | 0.7473 | 0.7533 | 0.63568 | 0.62998 | 0.66378 | 0.70363 | 0.9999995234784 |
| MEC_Desmarais | 0.68607 | 0.7473 | 0.75341 | 0.63568 | 0.6301 | 0.66366 | 0.70352 | 0.9999995241633 |
| MEC_Desmarais_duo | 0.5447 | 0.61779 | 0.62305 | 0.49016 | 0.48448 | 0.51993 | 0.56536 | 0.999949262 |

**Supplementary Table S8:** Number of alleles, number of effective alleles, information index, diversity, and unbiased diversity were determined by locus for the male and female Kurds using GenAIEx 6.5.

Kurds male

|  | **DXS9902** | **DXS10164** | **DXS7130** | **DXS7423** | **DXS8378** | **GATA172D05** | **DXS9898** | **DXS7424** | **GATA31E08** | **DXS6795** | **DXS981** | **DXS7132** | **GATA144D04** |
| --- | --- | --- | --- | --- | --- | --- | --- | --- | --- | --- | --- | --- | --- |
| **N** | 159 | 159 | 159 | 159 | 159 | 159 | 159 | 159 | 159 | 159 | 159 | 159 | 159 |
| **Na** | 5 | 7 | 9 | 5 | 6 | 8 | 6 | 9 | 7 | 6 | 7 | 5 | 7 |
| **Ne** | 3.096 | 2.837 | 3.417 | 3.152 | 3.042 | 5.360 | 3.521 | 4.710 | 4.735 | 3.194 | 3.338 | 3.696 | 3.688 |
| **I** | 1.204 | 1.242 | 1.569 | 1.297 | 1.224 | 1.775 | 1.404 | 1.783 | 1.641 | 1.303 | 1.421 | 1.402 | 1.500 |
| **h** | 0.677 | 0.648 | 0.707 | 0.683 | 0.671 | 0.813 | 0.716 | 0.788 | 0.789 | 0.687 | 0.700 | 0.729 | 0.729 |
| **uh** | 0.681 | 0.652 | 0.712 | 0.687 | 0.676 | 0.819 | 0.720 | 0.793 | 0.794 | 0.691 | 0.705 | 0.734 | 0.733 |

Kurds female

|  | **DXS9902** | **DXS10164** | **DXS7130** | **DXS7423** | **DXS8378** | **GATA172D05** | **DXS9898** | **DXS7424** | **GATA31E08** | **DXS6795** | **DXS981** | **DXS7132** | **GATA144D04** |
| --- | --- | --- | --- | --- | --- | --- | --- | --- | --- | --- | --- | --- | --- |
| **N** | 66 | 66 | 66 | 66 | 66 | 66 | 66 | 66 | 66 | 66 | 66 | 66 | 66 |
| **Na** | 4 | 7 | 10 | 5 | 6 | 7 | 5 | 8 | 7 | 5 | 5 | 5 | 7 |
| **Ne** | 2.903 | 2.389 | 3.136 | 3.098 | 3.061 | 5.425 | 3.799 | 4.252 | 4.639 | 3.151 | 2.717 | 3.236 | 4.020 |
| **I** | 1.128 | 1.142 | 1.434 | 1.278 | 1.242 | 1.751 | 1.425 | 1.638 | 1.610 | 1.329 | 1.192 | 1.275 | 1.605 |
| **Ho** | 0.591 | 0.576 | 0.727 | 0.818 | 0.667 | 0.848 | 0.652 | 0.894 | 0.773 | 0.667 | 0.591 | 0.697 | 0.727 |
| **He** | 0.656 | 0.581 | 0.681 | 0.677 | 0.673 | 0.816 | 0.737 | 0.765 | 0.784 | 0.683 | 0.632 | 0.691 | 0.751 |
| **uHe** | 0.661 | 0.586 | 0.686 | 0.682 | 0.678 | 0.822 | 0.742 | 0.771 | 0.790 | 0.688 | 0.637 | 0.696 | 0.757 |
| **F** | 0.099 | 0.010 | -0.068 | -0.208 | 0.010 | -0.040 | 0.116 | -0.169 | 0.015 | 0.023 | 0.065 | -0.009 | 0.032 |

| **Na = No. of Different Alleles** |
| --- |
| **Ne = No. of Effective Alleles = 1 / (Sum pi^2)** |
| **I = Shannon's Information Index = -1* Sum (pi * Ln (pi))** |
| **Ho = Observed Heterozygosity = No. of Hets / N** |
| **He = Expected Heterozygosity = 1 - Sum pi^2** |
| **uHe = Unbiased Expected Heterozygosity = (2N / (2N-1)) * He** |
| **F = Fixation Index = (He - Ho) / He = 1 - (Ho / He)** |
| **Where pi is the frequency of the ith allele for the population & Sum pi^2 is the sum of the squared population allele frequencies.** |

**Supplementary Table S9:** Pairwise p values of linkage disequilibrium test for all pairs of loci using 66 female samples from the Kurdish population.

| **Name** | **GATA31E0** | **GATA172D** | **GATA144D** | **DXS9902** | **DXS9898** | **DXS981** | **DXS8378** | **DXS7424** | **DXS7423** | **DXS7132** | **DXS7130** | **DXS6795** | **DXS10164** |
| --- | --- | --- | --- | --- | --- | --- | --- | --- | --- | --- | --- | --- | --- |
| **DXS10164** | 0.042554 | 0.619 | 0.366434 | 0.697226 | 0.225936 | 0.7468 | 0.547294 | 0.086734 | 0.288012 | 0.545692 | 0.070634 | 0.26752 | 0 |
| **DXS6795** | 0.208752 | 0.390578 | 0.88924 | 0.05275 | 0.779892 | 0.857076 | 0.647116 | 0.486354 | 0.146208 | 0.193286 | 0.799662 | 0 | 0.26752 |
| **DXS7130** | 0.461436 | 0.549644 | 0.0316 | 0.110032 | 0.00408 | 0.0001 | 0.452338 | 0.198398 | 0.510806 | 0.25229 | 0 | 0.799662 | 0.070634 |
| **DXS7132** | 0.329014 | 0.663576 | 0.348494 | 0.48426 | 0.806168 | 0.201824 | 0.588884 | 0.01535 | 0.848002 | 0 | 0.25229 | 0.193286 | 0.545692 |
| **DXS7423** | 0.206542 | 0.01191 | 0.278738 | 0.540816 | 0.756058 | 0.435576 | 0.118938 | 0.35357 | 0 | 0.848002 | 0.510806 | 0.146208 | 0.288012 |
| **DXS7424** | 0.193416 | 0.681618 | 0.035482 | 0.137216 | 0.951566 | 0.866332 | 0.206688 | 0 | 0.35357 | 0.01535 | 0.198398 | 0.486354 | 0.086734 |
| **DXS8378** | 0.841056 | 0.405656 | 0.00105 | 0.85157 | 0.426864 | 0.755138 | 0 | 0.206688 | 0.118938 | 0.588884 | 0.452338 | 0.647116 | 0.547294 |
| **DXS981** | 0.473298 | 0.03302 | 0.053628 | 0.255688 | 0.226872 | 0 | 0.755138 | 0.866332 | 0.435576 | 0.201824 | 0.0001 | 0.857076 | 0.7468 |
| **DXS9898** | 0.2134 | 0.105046 | 0.272746 | 0.072674 | 0 | 0.226872 | 0.426864 | 0.951566 | 0.756058 | 0.806168 | 0.00408 | 0.779892 | 0.225936 |
| **DXS9902** | 0.319542 | 0.765222 | 0.626212 | 0 | 0.072674 | 0.255688 | 0.85157 | 0.137216 | 0.540816 | 0.48426 | 0.110032 | 0.05275 | 0.697226 |
| **GATA144D** | 0.07274 | 0.808904 | 0 | 0.626212 | 0.272746 | 0.053628 | 0.00105 | 0.035482 | 0.278738 | 0.348494 | 0.0316 | 0.88924 | 0.366434 |
| **GATA172D** | 0.175386 | 0 | 0.808904 | 0.765222 | 0.105046 | 0.03302 | 0.405656 | 0.681618 | 0.01191 | 0.663576 | 0.549644 | 0.390578 | 0.619 |
| **GATA31E0** | 0 | 0.175386 | 0.07274 | 0.319542 | 0.2134 | 0.473298 | 0.841056 | 0.193416 | 0.206542 | 0.329014 | 0.461436 | 0.208752 | 0.042554 |

**Supplementary Table S10:** Pairwise p values of linkage disequilibrium test for all pairs of loci using 159 male samples from the Kurdish population.

| **Name** | **GATA31E0** | **GATA172D** | **GATA144D** | **DXS9902** | **DXS9898** | **DXS981** | **DXS8378** | **DXS7424** | **DXS7423** | **DXS7132** | **DXS7130** | **DXS6795** | **DXS10164** |
| --- | --- | --- | --- | --- | --- | --- | --- | --- | --- | --- | --- | --- | --- |
| **DXS10164** | 0.897674 | 0.420548 | 0.447602 | 0.214382 | 0.52629 | 0.859894 | 0.292796 | 0.762566 | 0.840062 | 0.119426 | 0.370532 | 0.115926 | 0 |
| **DXS6795** | 0.948478 | 0.170826 | 0.054578 | 0.163616 | 0.831548 | 0.65534 | 0.58754 | 0.714924 | 0.115202 | 0.265768 | 0.69577 | 0 | 0.115926 |
| **DXS7130** | 0.024228 | 0.962708 | 0.915038 | 0.859826 | 0.857752 | 0.476422 | 0.068482 | 0.499152 | 0.179514 | 0.63093 | 0 | 0.69577 | 0.370532 |
| **DXS7132** | 0.597892 | 0.83271 | 0.428974 | 0.719144 | 0.777808 | 0.933212 | 0.689342 | 0.721924 | 0.420486 | 0 | 0.63093 | 0.265768 | 0.119426 |
| **DXS7423** | 0.188728 | 0.679306 | 0.558646 | 0.33683 | 0.86949 | 0.012878 | 0.068606 | 0.508198 | 0 | 0.420486 | 0.179514 | 0.115202 | 0.840062 |
| **DXS7424** | 0.57959 | 0.019426 | 0.53834 | 0.573484 | 0.28188 | 0.746768 | 0.511494 | 0 | 0.508198 | 0.721924 | 0.499152 | 0.714924 | 0.762566 |
| **DXS8378** | 0.823448 | 0.713504 | 0.400892 | 0.261602 | 0.635158 | 0.271008 | 0 | 0.511494 | 0.068606 | 0.689342 | 0.068482 | 0.58754 | 0.292796 |
| **DXS981** | 0.987432 | 0.408616 | 0.28524 | 0.814964 | 0.050182 | 0 | 0.271008 | 0.746768 | 0.012878 | 0.933212 | 0.476422 | 0.65534 | 0.859894 |
| **DXS9898** | 0.398784 | 0.240892 | 0.013496 | 0.441726 | 0 | 0.050182 | 0.635158 | 0.28188 | 0.86949 | 0.777808 | 0.857752 | 0.831548 | 0.52629 |
| **DXS9902** | 0.486086 | 0.3504 | 0.13593 | 0 | 0.441726 | 0.814964 | 0.261602 | 0.573484 | 0.33683 | 0.719144 | 0.859826 | 0.163616 | 0.214382 |
| **GATA144D** | 0.89285 | 0.880148 | 0 | 0.13593 | 0.013496 | 0.28524 | 0.400892 | 0.53834 | 0.558646 | 0.428974 | 0.915038 | 0.054578 | 0.447602 |
| **GATA172D** | 0.093704 | 0 | 0.880148 | 0.3504 | 0.240892 | 0.408616 | 0.713504 | 0.019426 | 0.679306 | 0.83271 | 0.962708 | 0.170826 | 0.420548 |
| **GATA31E0** | 0 | 0.093704 | 0.89285 | 0.486086 | 0.398784 | 0.987432 | 0.823448 | 0.57959 | 0.188728 | 0.597892 | 0.024228 | 0.948478 | 0.897674 |
